# Regular Spiking and Intrinsic Bursting Pyramidal Cells Show Orthogonal Forms of Experience-Dependent Plasticity in Layer V of Barrel Cortex

**DOI:** 10.1016/j.neuron.2011.11.034

**Published:** 2012-01-26

**Authors:** Vincent Jacob, Leopoldo Petreanu, Nick Wright, Karel Svoboda, Kevin Fox

**Affiliations:** 1 School of Bioscience, Cardiff University, Cardiff CF10 3AX, UK; 2 Janelia Farm Research Campus, Howard Hughes Medical Institute, Ashburn, VA 20147, USA

## Abstract

Most functional plasticity studies in the cortex have focused on layers (L) II/III and IV, whereas relatively little is known of LV. Structural measurements of dendritic spines in vivo suggest some specialization among LV cell subtypes. We therefore studied experience-dependent plasticity in the barrel cortex using intracellular recordings to distinguish regular spiking (RS) and intrinsic bursting (IB) subtypes. Postsynaptic potentials and suprathreshold responses in vivo revealed a remarkable dichotomy in RS and IB cell plasticity; spared whisker potentiation occurred in IB but not RS cells while deprived whisker depression occurred in RS but not IB cells. Similar RS/IB differences were found in the LII/III to V connections in brain slices. Modeling studies showed that subthreshold changes predicted the suprathreshold changes. These studies demonstrate the major functional partition of plasticity within a single cortical layer and reveal the LII/III to LV connection as a major excitatory locus of cortical plasticity.

## Highlights

► Plasticity in layer V precedes plasticity in other cortical layers ► Regular spiking cells show spike-rate depression but not potentiation ► Intrinsic burster cells show potentiation but little depression ► Layer II/III to V connections show analogous RS/IB subtype-specific plasticity

## Teaser

Jacob et al. discuss the different adaptation of two subtypes of layer V cortical neuron to lasting changes in sensory input and reveal the LII/III to LV connection as a major excitatory locus of cortical plasticity.

## Introduction

Sensory experience shapes receptive field structure during distinct critical periods of development (Daw et al., 1992; Fox, 1992; Stern et al., 2001; Wiesel and Hubel, 1963). Changing the whisker complement alters receptive fields in the barrel cortex (Wallace and Fox, 1999) and altering visual input can change ocular dominance in the visual cortex (Wiesel and Hubel, 1963). These adaptive processes are thought to tune sensory neurons to the features they detect in the environment. In adulthood, plasticity persists in visual and somatosensory cortex chiefly in extragranular layers (LII/III and LV) (Daw et al., 1992; Diamond et al., 1994; Fox, 1992). Most of the functional studies on experience-dependent plasticity to date have either investigated plasticity in LIV or the superficial layers of cortex (LII/III), while relatively little is known of the functional plasticity in LV cells (Beaver et al., 2001; Diamond et al., 1994; Erchova et al., 2003; Wilbrecht et al., 2010). Conversely, most of the studies on structural plasticity to date have investigated spine plasticity of LV neurons (Hofer et al., 2009; Trachtenberg et al., 2002; Wilbrecht et al., 2010). LV is a major output projection layer of the cortex and in the somatosensory system sends connections to a variety of subcortical targets including trigeminal, pontine, thalamic, striatal, and collicular locations as well as other cortical areas (see Fox, 2008). The relative paucity of studies on LV plasticity makes it difficult both to relate spine plasticity to functional plasticity and to gain some understanding of how cortical plasticity affects intracortical circuits and subcortical targets.

LV contains a major subdivision between LVa and LVb and these layers are engaged by distinct cortical circuits (Manns et al., 2004; Schubert et al., 2006; Shepherd et al., 2005; Shepherd and Svoboda, 2005). Within LVb, pyramidal cells have diverse soma sizes, dendritic morphologies and synaptic targets (Chagnac-Amitai et al., 1990; Hattox and Nelson, 2007; Larkman et al., 1992; Mason and Larkman, 1990; Tsiola et al., 2003). The intrinsic bursting (IB) and regular spiking (RS) cells within LVb can be distinguished by their intrinsic firing patterns and their somatic and dendritic morphology (Agmon and Connors, 1992; Chagnac-Amitai et al., 1990; Zhu and Connors, 1999), although it has been argued that the morphological distinctions may represent two ends of a continuous spectrum rather than discrete categories of cell type. IB cells fire bursts of spikes in response to steady somatic current injection and tend to have complex dendritic arbors and large somata. RS cells fire adapting trains of spikes in response to steady current injection and tend to have relatively simple dendritic arbors and small somata. The intracortical circuits for IB and RS cells are different (Schubert et al., 2001) and IB cells project to thalamus, pons, and colliculus while RS cells project to cortical and striatal targets (Gao and Zheng, 2004; Le Bé et al., 2007). Spine plasticity also differs between the two cell types, with complex tufted cells showing greater spine plasticity in response to whisker deprivation than regular spiking cells (Holtmaat et al., 2006; Knott et al., 2006). Therefore, as a first step to understanding LV plasticity we studied plasticity in IB and RS cells.

We used extracellular spike recording to map the time course of cortical plasticity in LV of the barrel cortex of rats and mice followed by intracellular recording in vivo to measure plasticity in IB and RS cells characterized using their intrinsic firing properties. We used quantitative laser scanning photostimulation to map the circuits impinging on LVb neurons to understand the intracortical circuits contributing to the plasticity in LV. We found that plasticity was distinctly different between RS and IB cells and that the LII/III to V projections terminating on RS and IB cells are a major determinant of plasticity within the local cortical microcircuit.

## Results

### In Vivo Extracellular Single-Unit Recordings

#### Characterization and Time Course of Row Deprivation Induced Plasticity in Rats

We measured extracellular spike responses to whisker stimulation in order to obtain an overall picture of the time course of deprivation induced plasticity in different cortical layers. Animals were age P32–45 at the start of deprivation. We recorded receptive fields of 452 single cells in four layers at 3 time points in anaesthetized Long Evans rats.

**Figure 1:**
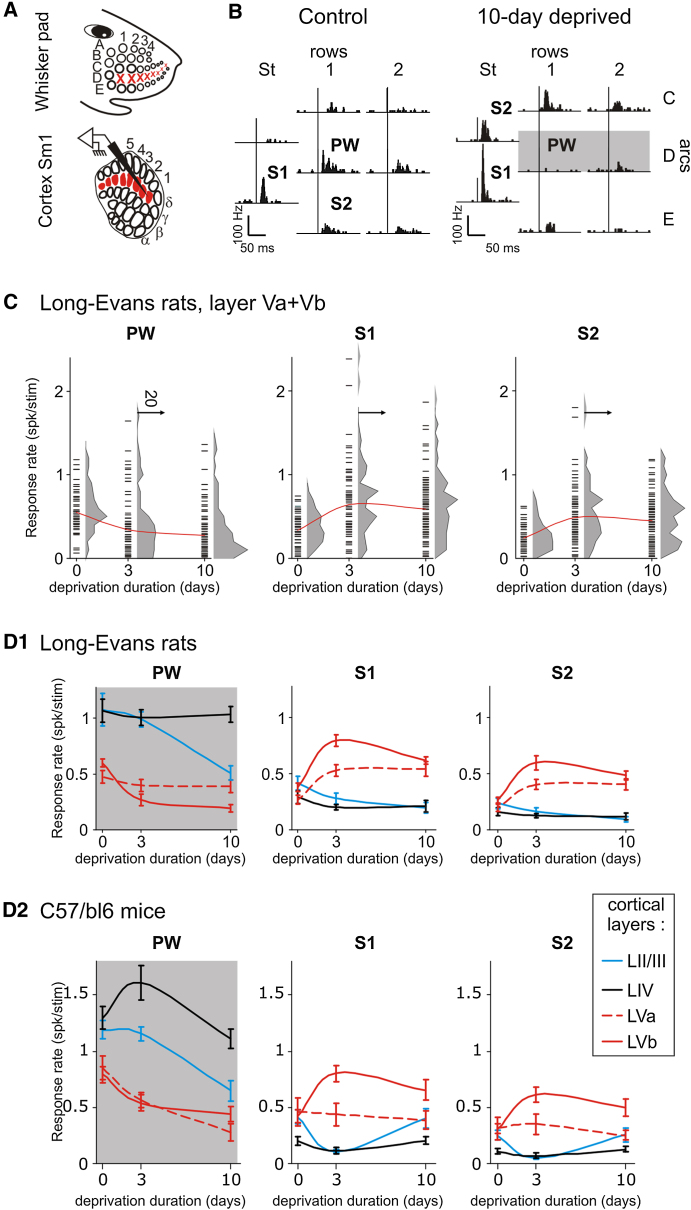
The Time Course of Receptive Field Plasticity in Different Layers (Rat and Mouse) (A) Pattern of whisker deprivation. The D row whiskers were removed from D1 to D8. Delta and Gamma were left intact. This produced a single row of deprived barrels in the cortex. We recorded from the D1, D2, and D3 deprived barrel-related columns. (B) Poststimulus time histograms were constructed and analyzed for responses to stimulation of all whiskers immediately surrounding the principal whisker. Control: an example receptive field for a LV cell in an undeprived animal. Right: A typical LV receptive field in an animal deprived for 10 days. The shading indicates PSTHs corresponding to deprived whiskers. S1 and S2 refer to the largest and second largest responses respectively produced by surround whiskers. (St = whisker straddling the rows, i.e., gamma and delta). (C) Distribution of layer V responses to principal whisker (PW), S1 and S2 surround whiskers. For each deprivation length, response amplitude is plotted for each individual cell (black symbols) and the population distribution is shown at the side (shaded area, arrows: 20 cells for 0.05 spk/stim-wide bins). Red lines: response average versus time-course. (D) Time course of PW, S1, and S2 responses are plotted for rats (D1) and mice (D2) against the numbers of days of D-row deprivation. For rats, PW: note that LIV and LVa responses do not change over this period. LII/III PW responses are lower at 10 days but not 3 days. LVb responses decrease after 3 days deprivation. S1 and S2: LIV and LII/III responses do not change while LVa and Vb responses potentiate after 3 days and 10 days deprivation. See results for statistics. See also Tables S1 and S2.

Principal whisker responses were affected by D-row deprivation over the 10 day time course only in LII/III and Vb (Figure 1). A two-way ANOVA showed an effect of layer (F(3,3) = 66.5, p < 0.0001) and deprivation time (F(2,2) = 13.0, p < 0.0001) and interactions between deprivation and layer (F(6,6) = 3.7, p < 0.002) (see Table S1 available online for all post hoc t tests). LVb neurones located in deprived barrels showed the earliest depression of principal whisker responses before any other layer was affected, showing a reduction to 52% of control levels after just 3 days deprivation (t_(71)_ = 5.3; p < 0.001). After 10 days of D-row deprivation, depression was also observed in layers II/III (reduction to 60% of control levels, t_(54)_ = 3.1; p < 0.01). Principal whisker responses of neurones in layers IV and Va were unaffected by deprivation at any time point (two-way ANOVA, no effect of deprivation F(2,2) = 0.45, p = 0.636, nor interaction between deprivation and layer F(2,2) = 0.05, p = 0.95).

We also characterized the responses to stimulation of the spared row whiskers for the same cells. Responses of spared surround whiskers were ordered from greatest to smallest for each cell (i.e., S1, S2, … S8) before averaging the responses across cells. The only cells showing clear and significant potentiation in response to D-row deprivation were located in LVa and Vb (Figure 1). A two-way ANOVA for the strongest S1 whisker response showed an effect of layer (F(3,3) = 29.2, p < 0.0001), deprivation (F(2,2) = 4.37, p < 0.02) and an interaction between layer and deprivation (F(6,6) = 7.46, p < 0.0001) and similarly for S2: layer F(3,3) = 43.3, p < 0.0001, deprivation F(2,2) = 6.9, p < 0.001, interaction F(6,6,) = 7.0, p < 0.0001) (see Table S1 for all post hoc t tests). In LVa, the S1 and S2 responses increased almost two fold at 3 days (197% and 205%, respectively) and maintained that level at 10 days (203% and 206% of control values), which was highly statistically significant (for S1, t_(60)_ = 2.95, p < 0.005 and for S2 t_(60)_ = 3.0, p < 0.004). In LVb, the S1 response also increased by about 2-fold at 3 days (to 201% of control) and the S2 response by 225% of control values and while both fell back slightly after 10 days of deprivation (to 163% and 183% for S1 and S2, respectively), they were still highly significantly greater than control values in both cases (for S1 t_(91)_ = 4.0, p < 0.0001 and for S2, t_(91)_ = 4.2, p < 0.0001).

#### Comparison of the Time Course of Deprivation between Mice and Rats

We repeated our experiments in mice to see whether the findings would generalize. We studied the receptive fields of 474 cells over identical D-row deprivation conditions (Figure 1D). Mice had slightly stronger principal whisker responses and slightly smaller surround whisker responses. Receptive field kurtosis was greater in mouse than rat in LIV (5.8 versus 5.16), LVa (3.1 versus 2.6), and LVb (2.4 versus 2.2), but not in LII/III (3.6 versus 3.8).

With the notable exception of layer Va, the reaction of cells in the different cortical layers to D-row deprivation was almost identical in the two species and in particular for the main effects described for layers Vb and LII/III above (Figure 1; see Table S2 for all similarities and differences). The S1 whisker response potentiated after 3 days in mice in LVb and was the only layer to show any potentiation (Figure 1D). The response increased 190% at 3 day and 155% at 10 days both of which were highly significant (t_(98)_ = 4.1, p < 0.0001; t_(82)_ = 2.3, p < 0.03). The LII/III surround whisker responses showed no potentiation in either species, but the principal whisker response depressed in LII/III in mice at 10 days but not at 3 days similar to the rat (for 3 day time point t_(127)_ = 0.35, p = 0.73; at 10 days, t_(94)_ = 4.2, p < 0.0001). The main difference between the rat and mouse results was the plasticity in layer Va. We did not observe any potentiation of the S1 or S2 whisker response in LVa in mice whereas this effect was clear in rats (Figure 1D; Table S2). Conversely, the principal whisker showed only a minor and statistically insignificant depression in layer Va of the rat, but was clearly depressed in the mice (Figure 1D; Table S2).

### In Vivo Intracellular Recordings

#### Cell-Type Analysis and Receptive Field Mapping

**Figure 2:**
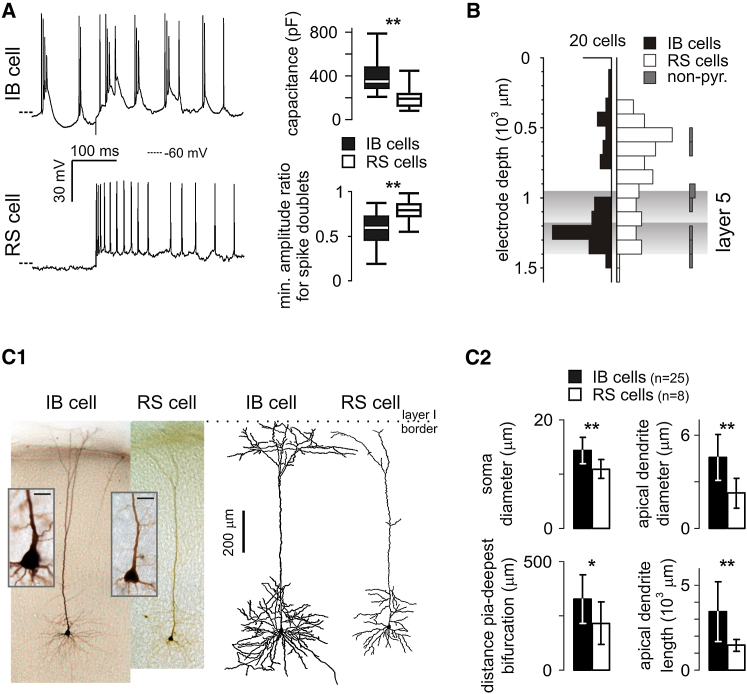
In Vivo Classification of Pyramidal Cell Types (Rat) (A) Electrophysiological characterization. Left, examples of firing pattern in response to current injection used for classification. Right, quartile representation of distribution of LV electrophysiological parameters recorded during stimulation. Cell capacitance was calculated from double exponential fits of membrane response to negative current (10 pA, 0.2 Hz) injected in-between stimulation sequences. The minimum ratio of spike amplitude was calculated from evoked spike doublets. Kolmogorov-Smirnov test, ^∗∗^p < 10^−6^. (B) Laminar distribution of the different cell types. Only LV IB and RS cells were considered in subsequent analysis. (C) Morphological analysis of IB and RS cells. (C1) Left, examples of biocytin-filled cells. Inset: zoom along the cellular soma for the same two cells (bar: 20 μm). Right: dendritic tree reconstruction for the two cells corresponding to the firing patterns presented in (A). (C2) Cell type analysis of morphologic parameters (mean ± SD). The width of the apical dendrite was calculated 10 μm away from the point where the dendrite emerges from the soma. Unpaired Student's t test, ^∗^p < 0.05; ^∗∗^p < 0.005. See also Figure S4.

We concentrated on layers Va and Vb in the rat and used intracellular recording with sharp electrodes in order to identify RS and IB pyramidal cell subtypes (Figure 2A; see Experimental Procedures). Animals were age P32–45 at the start of deprivation and recorded at 10 days after the start of deprivation. In agreement with the literature (Nowak et al., 2003), RS cells (33 cells) lay uniformly in both LVa and Vb (Kolmogorov-Smirnov test for depth uniformity, p > 0.6) while IB (39 cells) cells were preferentially located in LVb (Kolmogorov-Smirnov test for depth uniformity, p < 0.01; Figure 2B). IB cells had higher capacitances (Figure 2A; sum of rank test p < 10^−7^) and displayed a stronger spike amplitude adaptation than RS cells (sum of rank test p < 10^−6^). Cell morphology was recovered for a subset of cells characterized by firing pattern and receptive field together with cells for which the receptive field was not determined (Figure 2C). From this data set, we found that IB cells and RS cells differed in soma diameter (t_(31)_ = 3.80, p < 0.001), apical dendrite diameter (t_(31)_ = 4.03, p < 0.0005), ratio between apical dendrite diameter and soma diameter (t_(31)_ = 3.86, p < 0.001), distance between the pia and the deepest bifurcation (t_(30)_ = 2.54, p < 0.02), and total apical dendrite length (t_(30)_ = 3.33, p < 0.005). These observations are consistent with the idea that IB cells are likely to correspond to thick tufted and RS cells to thin slender pyramidal neurons (Chagnac-Amitai et al., 1990; Gao and Zheng, 2004; Le Bé et al., 2007). Average membrane potential and resistance did not differ between control and deprived animals, neither for IB cells (respectively, Vm = −64.0 ± 5.1 versus 64.1 ± 8.4 mV and R = 28.4 ± 12.9 versus 27.3 ± 14.7 MΩ) nor for RS cells (Vm = −61.3 ± 4.9 versus −63.0 ± 5.5 mV and R = 28.8 ± 10.7 versus 22.3 ± 12.1 MΩ). In control animals, IB cells had greater whisker responses compared to RS cells (two-way ANOVA, F(1,1) = 24.6, p < 10^−5^), in agreement with (de Kock et al., 2007).

#### RS and IB Cell Input Receptive Fields Show Inverse Complementary Forms of Plasticity

**Figure 3:**
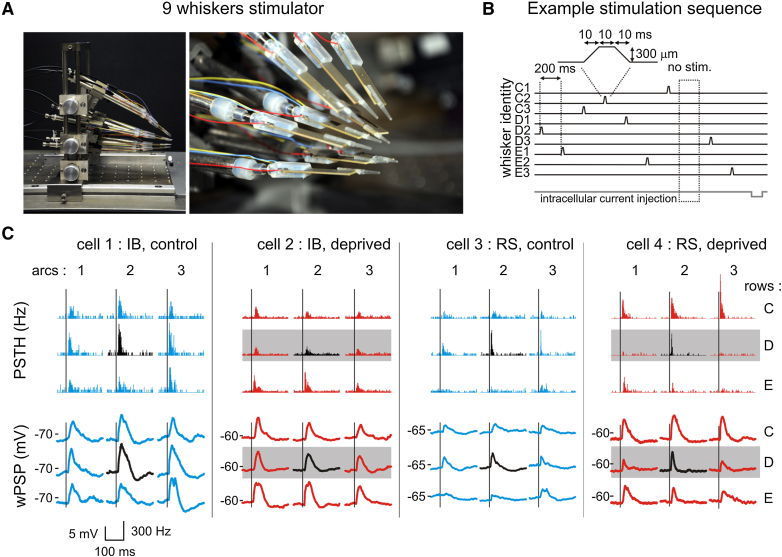
Stimulation Methodology and Receptive Field Case Studies (Rat) (A) Pictures of the 3 × 3 whisker stimulation device. Each piezo is oriented at the natural angle of the whisker unless stimulated. (B) Schematic representation of one sequence of stimulation applied for recordings in the D2 column. For D1 or D3 recordings, the stimulators are translated so that the PW is always the central whisker. For each sequence the whiskers are stimulated with a dorsal deflection in a random order. An intracellular injection of current is made at the end of each sequence (0.1 pA, 100 ms). (C) Nine-whisker suprathreshold (PSTH) and subthreshold (wPSP) receptive fields for 4 example cells. Relative positions map the spatial arrangements of the whiskers. Vertical lines indicate stimulus onset. Black PSTH and wPSP correspond to the principal whisker (D2 in the four cases). The shaded areas highlight the responses to deprived whiskers stimulation. See also Figure S5.

We used sparse noise stimuli applied via a nine-whisker stimulator (Jacob et al., 2010) to map receptive fields (Figures 3A and 3B). Examples of LV receptive fields evaluated using peristimulus time histograms (PSTH) and whisker-evoked postsynaptic potentials (wPSP) are shown in Figure 3C (neurons 2 and 4 are D-row deprived).

We found that RS and IB cells' suprathreshold receptive fields were affected differently by whisker deprivation: an ANOVA for all whisker responses confirmed a significant interaction between deprivation and cell type (F(1,1) = 5.1, p < 0.05). This was because the deprived whisker responses were depressed for RS cells (F(1,1) = 13, p < 0.001) but not for IB cells (F(1,1) = 1.0, p > 0.3), whereas spared whiskers responses were potentiated for IB cells (F(1,1) = 5.6, p < 0.02) but not for RS cells (F(1,1) = 0.2, p > 0.6). Therefore, while the in vivo intracellular recordings showed the same overall potentiation and depression components seen in the extracellular studies, remarkably the potentiation and depression components were split between IB and RS cells, respectively.

**Figure 4:**
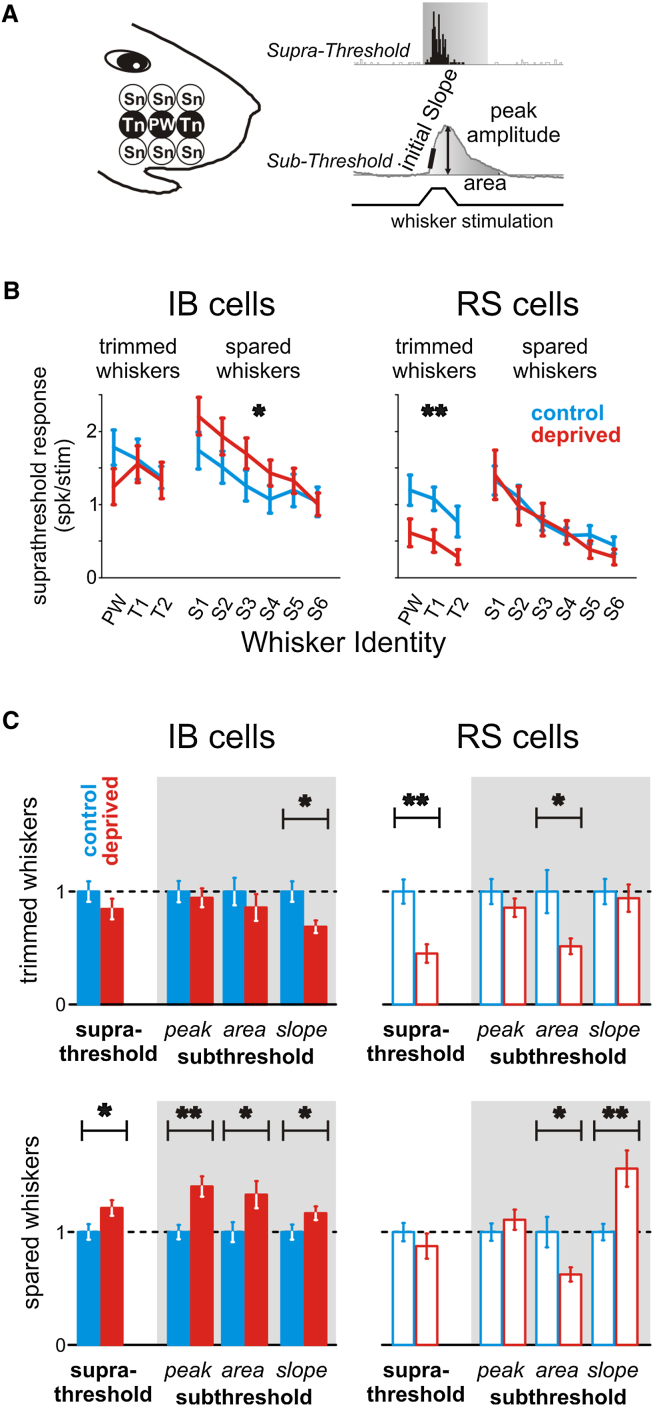
Deprivation Induces Cell-Type-Specific Changes of LV Receptive Fields (Rat) (A) Left, schematic representation of the whiskers studied; right, illustration of the calculated parameters. (B) Suprathreshold receptive field. Principal whisker and two surround row-associated whiskers (T1 and T2) were trimmed, while six surround whiskers in the adjacent rows (S1 to 6) were left intact. Trimmed and spared surround whiskers were ordered respectively in decreasing order of suprathreshold response (mean ± SEM). Test: ANOVA, effect of deprivation on all spared or all trimmed whiskers; ^∗^p < 0.05, ^∗∗^p < 0.001. (C) Supra and subthreshold effects of deprivation on all trimmed whiskers averaged and all spared whiskers averaged. Each parameter is normalized to the control value. The subthreshold parameters (shaded areas) are calculated from the wPSPs. Receptive fields are detailed as in (B) for each parameter in Figure S1. Same statistics as in (B). See also Figures S1 and S5.

To understand the derivation of the suprathreshold responses, we analyzed the time course of the wPSPs. The amplitude of the first wPSP peak, the total area of the whisker-evoked depolarization and the initial slope of the wPSP were calculated for RS and IB cells (all significant differences found are shown in Figure 4C and detailed further in Figure S1). Potentiation of the spared whisker responses in IB cells was reflected by an increase in all three parameters (peak: F(1,1) = 16.1, p < 0.0001; area: F(1,1) = 5.1, p < 0.05; slope: F(1,1) = 5.0, p < 0.05) and therefore corresponded in a simple manner with the suprathreshold responses. However, the initial slope of the wPSP was depressed for the deprived whisker response of the IB cells (F(1,1) = 6.7, p < 0.02) without an apparent concomitant change in the suprathreshold response (Figures 4B and 4C).

Depression of the deprived whisker responses in RS cells was reflected in a decrease in the area of the wPSP depolarization (F(1,1) = 5.8, p < 0.02). This was the only parameter that changed for the deprived whisker response and shows that a change in area of the wPSP is sufficient for a decrease in surprathreshold response. Consistent with this idea, the IB cells showed no decrease in area and no decrease in surprathreshold response.

The changes in the subthreshold responses to spared whisker stimulation for the RS cells were more complex and to some extent cancelled each other out. While the slope of the wPSP increased significantly (F(1,1) = 11.4, p < 0.001) the wPSP area decreased significantly (F(1,1) = 6.6, p < 0.02). While this has implications for the timing of the response as described in the next section, it had no overall effect on the suprathreshold responses (Figure 4B).

The initial slope of the wPSP reflects the activity of the first inputs to activate the cell following whisker stimulation and was correlated with the early but not the late evoked spikes (early: <15 ms after stimulation, linear regression from PW response r^2^ = 0.17, p < 0.001; late: >15 ms after stimulation, r^2^ = 0.03, p > 0.15). In contrast, the area of the wPSP was correlated with the late (r^2^ = 0.14, p < 0.002) but not the early evoked spikes (r^2^ = 0.02, p > 0.2). The wPSP amplitude peak occurred on average at 12 ms after stimulation for the PW and 18ms for S1 and was best correlated with the total spike count (r^2^ = 0.22, p < 10^−4^). Further analysis revealed that deprivation produced corresponding changes in wPSPs slope and early evoked spikes on the one hand and between depolarization area and late evoked spikes on the other (Figure S1). Indeed, an increase in wPSPs slope and a decrease in depolarization area for RS cells corresponded to a concomitant increase of the early component (F(1,1) = 5.3, p < 0.05) and decrease of the late component (F(1,1) = 3.9, p < 0.05) of the suprathreshold response. A decrease in the initial slope for the IB cells appeared to correspond to a decrease in the early component of the suprathreshold response, but this was not statistically significant (F(1,1) = 3.9, p = 0.051; Figure S1). Because the early or late components of the response are only fractions of the total response, significant changes in either component could be diluted (as with the trimmed whisker responses in IB cells) or cancelled out because they changed in opposite directions (as for spared whisker responses in RS cells) so that total spike count was not affected significantly.

We found that a simple integrate and fire model applied to the subthreshold activity was sufficient to explain deprivation-induced changes in suprathreshold activity. The membrane voltage distribution was shifted by the amount of depolarization (method 1) or recalculated for each time step around stimulation (method 2). The probability of firing was then calculated and used to create a predicted PSTH, which could be compared to the real PSTHs (see Figure S2). The exact shapes of the PSTHs were not closely reproduced for IB cells, but the effects of sensory deprivation were. The complex time-specific modifications observed for RS cells were also reproduced by the model. In conclusion, changes observed in suprathreshold activity directly reflected changes at the subthreshold level, which implies that synaptic and not spike generation mechanism explain the observed plasticity.

#### The Time Course of the wPSP Is Altered by Deprivation

**Figure 5:**
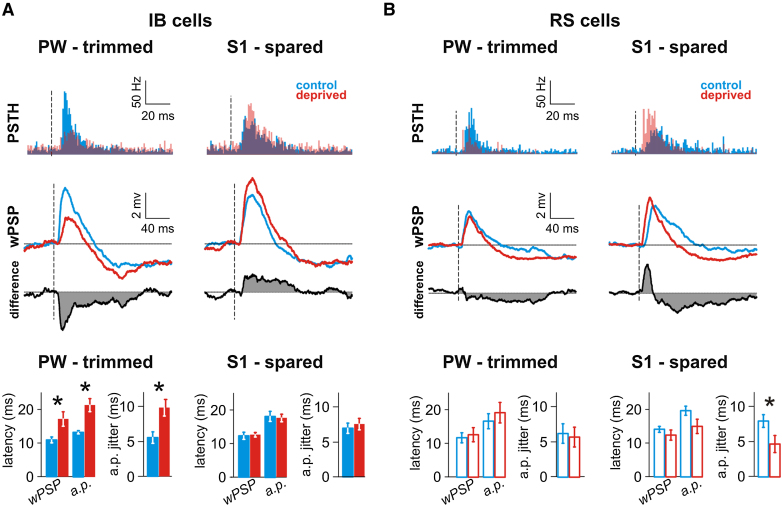
Temporal Properties of the Receptive Fields Are Differentially Affected by Deprivation (Rat) (A) IB cells response in control and deprived animals. Top: IB cell-population PSTH and wPSP for the principal and for the best spared whiskers in control and test animals. The average Vm after the no-stimulation event has been subtracted, and the baseline periods aligned. The difference between control and test wPSP is presented in gray. Dashed line: onset of stimulation. Bottom: Quantification of temporal parameters (mean ± SEM). The wPSP latency corresponds to the beginning of the depolarization, the a.p. latency is the averaged timing of the first spike following stimulation and the a.p. jitter is the standard deviation of the first spike timing. Unpaired Student's t test, ^∗^p < 0.05. (B) RS cell population PSTH and wPSP for the principal and for the best spared whiskers in control and test animals. Conventions as in (A). See also Figure S2.

As whisker deprivation affected the timing as well as the amplitude of the responses to whisker stimulation, we analyzed the time course of response for RS and IB cells. Figure 5 represents averaged wPSPs and PSTHs for one trimmed and one spared whisker only. Note that the early spikes (<15 ms poststimulus) were significantly depressed for PW stimulation in the IB cell population (t_(38)_ = 3.2, p < 0.005; see Figure S1), but not for the other trimmed whiskers, as revealed in Figure 5A. The potentiation in IB cells and the depression in RS cells appear to be roughly uniform throughout the whole time course of the response. In contrast, depression of PW responses in IB cells was greater in the early part of the response (<30 ms poststimulus). Similarly for RS cells, the best spared whisker was potentiated for the early part of the response (<25 ms poststimulus) but depressed for the late component. The latter observation held when all the spared whiskers were considered. We quantified the area of the subthreshold response in four time windows (0–25; 25–50; 50–75; 75–100 ms poststimulus) and the suprathreshold response in three time-windows (0–15; 15–30; 30–45 ms poststimulus). RS cells' response to spared whisker stimulation displayed a significant interaction between deprivation and time both for suprathreshold (F(2,2) = 6.8, p < 0.005) and subthreshold (F(3,3) = 5.5, p < 0.005) parameters. These data suggest that the early component of the response can be potentiated (as with the RS cells) or depressed (as with the IB cells) independently of later components of the wPSP.

Latency and jitter of evoked action potentials are important temporal parameters for coding sensory information. Figure 5 shows sub- and suprathreshold latency and jitter of the first action potential following stimulation for the PW and S1 (see Figure S1 for all whiskers). Both RS and IB cells' latency and jitter were affected by deprivation in an inverse complementary way. For IB cells, latency (F(1,1) = 16.6, p < 2.10^−4^) and jitter (F(1,1) = 11.2, p < 0.002) of the suprathreshold response to the deprived whiskers were increased, but were unchanged for the spared whiskers (latency, F(1,1) = 0.18, p > 0.6; jitter, F(1,1) = 0.001, p > 0.9). For RS cells, deprivation did not affect the temporal information in the response to stimulation of the deprived whiskers (latency, F(1,1) = 1.04, p > 0.3; F(1,1) = 0.006, jitter, p > 0.9), but both latency (F(1,1) = 7.6, p < 0.01) and jitter (F(1,1) = 15.0, p < 0.001) of action potentials evoked by deflections of the spared whiskers were decreased. Action potential rate and jitter can therefore change independently and did so in opposite directions for the different inputs to RS and IB cells.

Finally, the rate of spontaneous activity preceding stimulation was also affected by deprivation (Figure 5). We observed a significant decrease for RS cells (10.6 ± 2.2 versus 3.9 ± 1.6 Hz, t_(30)_ = 2.5, p < 0.05) but not for IB cells (11.8 ± 1.9 versus 16.0 ± 2.3 Hz, t_(38)_ = 1.4, p > 0.1).

To understand which intracortical pathways might give rise to these different components of plasticity, we studied the synaptic responses of LV neurons in whisker deprived mice ex vivo using laser scanning photo stimulation. As LVa showed no potentiation in mice we concentrated the study on LVb.

### Ex Vivo Laser Scanning Photostimulation and Whole-Cell Recordings

#### Input Maps of LVb Neurons

**Figure 6:**
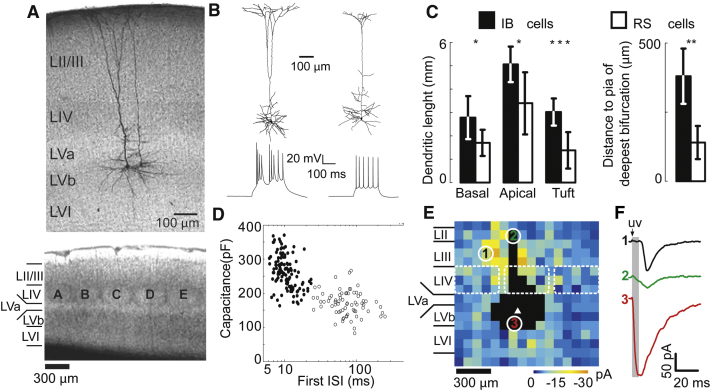
Measuring Synaptic Input Maps in Intrinsically Bursting and Regular Spiking Pyramidal Neurons (Mouse) (A) Top: Two biocytin-filled neurons in LVb (left, IB cell; right, RS cell). Bottom: Brightfield image of an acute brain slice showing the five rows of barrels. (B) Examples of dendritic morphologies (left, IB cell; right, RS cell) and the corresponding action potential firing patterns. (C) Quantitative morphology of the dendritic arbors of RS (n = 7) and IB (n = 7) cells. (D) First interspike interval (First ISI) versus membrane capacitance (solid circles, IB cell; open circles, RS cell). Cells with First ISI < 25 ms were classified as IB. (E) Example of a LSPS input map for a single LVb neuron (soma position is indicated by the triangle). Dashed boxes indicate barrel boundaries. The colored numbers in white circles indicate the locations of the UV stimulus generating the traces shown in (F). Pixels with direct responses are blacked out (see Experimental Procedures). (F) Examples of traces from the map shown in (E). 1, pure synaptic response; 2, direct response in the apical dendrite; 3, direct response in the perisomatic region. The gray zone represents the time used to score direct responses. See also Figures S3 and S4.

We analyzed barrel cortex circuits in brain slices cut across barrel rows (Allen et al., 2003; Finnerty et al., 1999) (Figure 6). In selected slices, five large barrels corresponding to barrel rows A–E could be identified under brightfield illumination (Figure 6A). LVb pyramidal neurons were distinguished by their firing patterns in response to threshold injection of current (Figures 6B and 6D; see Experimental Procedures). The dendrites of a subset of recorded neurons were reconstructed for morphological analysis (Figures 6A, 6B, and 6C). Again, we observed that IB cells had thick apical dendrites with a dominant bifurcation in LII/III or LIV (>230 μm below the pia) and an elaborate apical tuft. RS cells had a relatively thin apical dendrite and a small apical tuft branching close to the pia (<230 μm below the pia). IB cells also had larger membrane capacitances (Figure 6D; 264 ± 48 versus 175 ± 34 pF, rank sum test p < 5.10^−4^), higher resting potential (−68 ± 3 versus −70 ± 5 mV, rank sum test p < 0.005) and lower membrane resistances (127 ± 48 versus 201 ± 80 MΩ, rank sum test p < 5.10^−4^) than RS cells. Average membrane potential and resistance did not differ between control and deprived animals, neither for IB cells (respectively, Vm = −68.7 ± 3.7 versus −68.2 ± 3.6 mV and R = 142.3 ± 53.5 versus 124.4 ± 54.5 MΩ) nor for RS cells (Vm = −72 ± 4.8 versus −70 ± 5.1 mV and R = 229.2 ± 118.4 versus 164.7 ± 58 MΩ).

**Figure 7:**
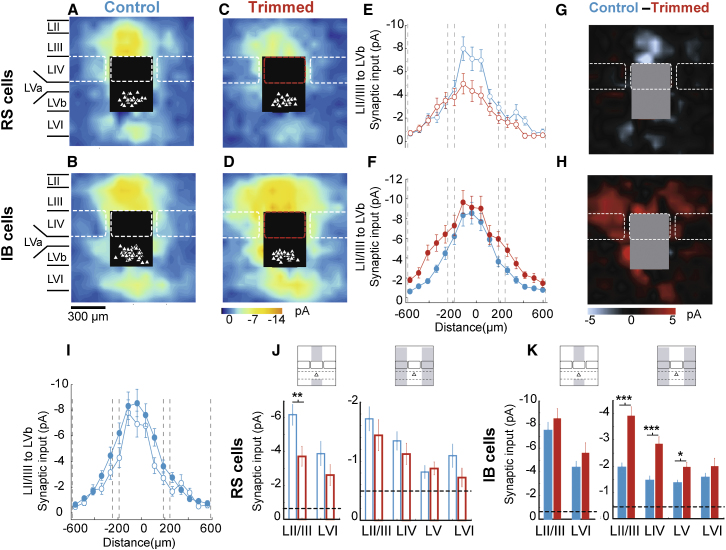
Plasticity of Synaptic Input Maps for RS and IB Cells in Deprived Barrel Columns (Mouse) (A and B) Average synaptic input maps of RS (n = 26) and IB (n = 46) cells under control conditions. The positions of barrels are indicated by dashed lines. The positions of the somata are indicated by white triangles. The areas in black indicate the regions where the number of traces not polluted by direct responses was too small and were excluded from analysis. (C and D) Average synaptic input maps of RS (n = 22) and IB (n = 33) cells in deprived barrel columns. The positions of spared (white) and deprived (red) barrels are indicated by dashed lines. (E and F) Horizontal spatial profiles of mean LII/III → LVB input to RS (E) and IB (F) cells in control (blue) and trimmed (red) animals. Dashed lines indicate the boundaries of the barrel columns. (G and H) Difference maps made by subtracting the averaged trimmed map (C and D) from the control map (A and B). (I) Horizontal spatial profiles of mean LII/III to LVb input to RS (open circles) and IB (filled circles) cells in control conditions. (J and K) Mean synaptic input sorted by layer of origin from the home (left) or surround columns (right) (indicated as gray boxes in the schematics) to RS (J) and IB (K) cells, in control (blue) and trimmed (red) animals. Background due to spontaneous synaptic currents is indicated by dashed lines. See also Figure S3.

To map the strength of excitatory projections converging onto LVb neurons we used laser scanning photostimulation (LSPS) by glutamate uncaging combined with whole cell recordings (Briggs and Callaway, 2005; Katz and Dalva, 1994; Shepherd et al., 2003; Shepherd and Svoboda, 2005; Yoshimura et al., 2005) (Figures 6E and 6F). Using LSPS, we mapped excitatory projections onto LVb neurons in an area encompassing three barrel columns (Figure 6E) and observed results in agreement with the literature (Briggs and Callaway, 2005; Hooks et al., 2011; Lefort et al., 2009; Schierloh et al., 2003; Schubert et al., 2001; Thomson and Bannister, 2003). Both RS and IB cells received input from all the cortical layers (Figures 7A and 7B) with a prominent LII/III to LVb projection (Figure 7). The majority of input from LII/III and LVI (the two layers where we could analyze both the home and surround columns) came from the home barrel column (68% ± 12%). Both cell types received smaller but significant input from all the layers in the neighboring barrel columns. IB cells had slightly broader input maps, receiving more transcolumnar input than RS cells, especially from the subgranular layers (LV p < 0.005; LVI p < 0.05) (Figures 7J and 7K).

#### RS and IB Cell Input Maps Show Complementary Forms of Plasticity

We induced experience-dependent plasticity in the barrel cortex by trimming a single row of whiskers (row C or D) so that the deprived barrel column was flanked on both sides by spared barrel columns. Animals were aged P30 at the start of deprivation. In brain slices from animals trimmed for 10–14 days we again measured the input maps for IB and RS neurons in deprived columns and compared them to input maps from controls. Significant experience-dependent changes in input maps of LVb neurons were seen in LII/III, LIV, and LV (Figures 7C, 7D, 7J, and 7K), but experience-dependent changes were most robust in the LII/III to LVb projection (Figures 7E, 7F, 7J, and 7K) both in RS and IB cells.

In spite of having similar input maps under control conditions, input maps of RS and IB cells changed in inverse complementary ways in response to whisker trimming (Figures 7E and 7F). The LII/III to LVb RS projection was reduced within the home column (60% ± 44% of control, p < 0.005) (“center depression”), while inputs from the surrounding barrel columns remained unchanged (86% ± 72%, p > 0.39) (Figures 7E, 7G, and 7J). In contrast, inputs to LVb IB neurons within the home column remained unchanged (LII/III, 114% ± 61%, p > 0.20; LVI 128% ± 111%, p > 0.32), while input from the surrounding barrel columns increased (LII/III, 201% ± 102%, p < 0.00005; LIV, 198% ± 104% p < 0.0001; LV, 145% ± 76% p < 0.008) (“surround potentiation”) (Figures 7F, 7H, and 7K). The excitatory projections to IB and RS neurons thus change in orthogonal patterns in response to whisker trimming.

**Figure 8:**
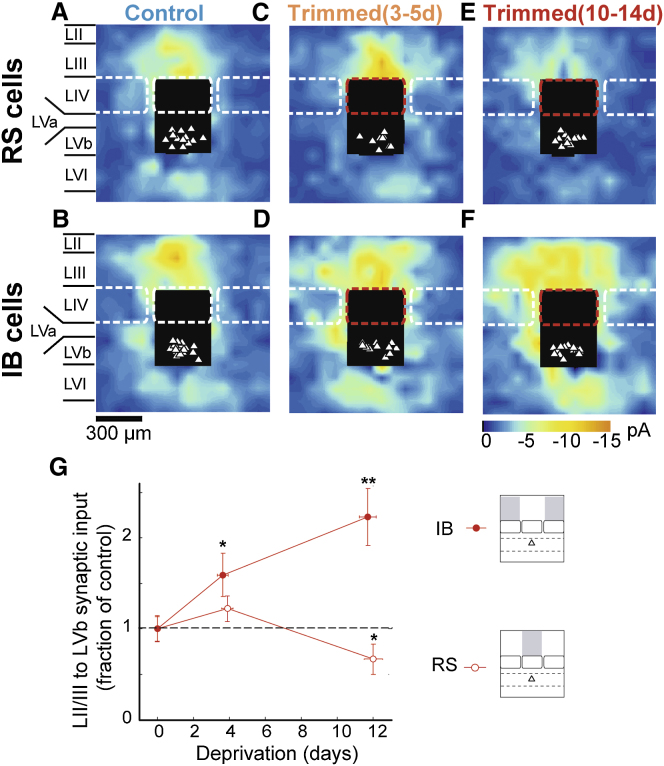
Time Course of the LII/II to LVb RS Projection Center Depression and the LII/III to LVb IB Projection Side Potentiation (Mouse) (A and B) Averaged synaptic maps of RS (n = 13) and IB (n = 18) cells under control conditions. (C and D) In deprived barrels, 3 to 5 days after whisker trimming (RS: n = 10; IB:n = 13). (E and F) In deprived barrels 10 to 14 days after whisker trimming (RS: n = 13; IB:n = 16). (G) Time course of LII/III to LVb RS input from the home barrel (open circles), and LII/III to LVb IB input from the surround columns (filled circles), normalized to controls. See also Figures S3 and S8.

The postsynaptic response to photostimulation (Q) depends on the average strength of the synaptic connection with the stimulated presynaptic neuron (qcon, defined here as the postsynaptic charge per presynaptic neuron per action potential), the number of neurons stimulated, and the number of action potentials fired per stimulated neuron (N_AP_). To confirm that experience-dependent changes in input maps can be attributed to synaptic plasticity (i.e., changes in qcon) we characterized the photoexcitability of presynaptic neurons before and after deprivation by recording excitation profiles (Shepherd et al., 2003; Shepherd and Svoboda, 2005). The average number of action potentials per neuron elicited by UV uncaging (N_AP_) was not affected by sensory deprivation (Figure S3). Similarly, the distance from the soma from which neurons could be excited, which together with N_AP_ determines the number of neurons photostimulated, was unchanged by deprivation. The deprivation-induced changes observed in the input maps therefore reflect synaptic changes. We measured changes in input maps with varying time between whisker trimming and LSPS mapping (Figure 8). Surround-potentiation in IB cells could already be detected 3–5 days after trimming and increased monotonically with time, but center-depression in RS cells could only be observed in animals deprived for 10 days or longer (Figure 8G). Deprived barrel input in IB cells and spared barrel input to RS cells remained at control levels at all deprivation lengths (Figure S6).

## Discussion

We used three different experimental methods to characterize cell type specific plasticity in LV and the underlying changes in cortical connectivity. We find that plasticity in LV precedes changes in other cortical layers, that potentiation and depression are decoupled in spatially overlapping RS and IB subpopulations within LV, and that changes in LII/III to LV excitatory connectivity mirror changes in stimulus-evoked action potential rate within the sensory-evoked response. All three methods are unanimous in showing the rapidity and independence of LV plasticity. Both intracellular studies are consistent in showing the striking parcellation of potentiation in IB cells and depression in RS cells. Furthermore, by comparing differences in plasticity revealed by whisker stimulation and direct stimulation of cortical circuit elements we can deduce that plasticity occurs in subcortical projections to LV, that they differ between RS and IB cells and that they are more concerned with the timing of sensory information than the strength of drive to the circuit.

### Relative Contribution of LII/III to V Pathways to Receptive Field Plasticity

Our main finding is that changes in the LII/III to Vb pathway within and between columns can explain most of the receptive field plasticity observed in vivo as well as the differences between RS and IB cells. The magnitude of potentiation seen in the three different experimental methods used here are strikingly similar. After 10 days of whisker trimming, the strength of cross-columnar LII/III to LVb projections measured ex vivo and stimulus evoked firing rates for spared whiskers measured in vivo both increased by a factor of 2. This suggests that potentiation of the cross-columnar LII/III to LVb connections is sufficient to explain changes in the IB cells' responses to spared whisker stimulation.

Depression of deprived pathway responses can also be explained by a depression of the LII/III to LVb synapses onto RS cells within the column from data obtained in the in vivo and ex vivo recordings. In both cases, the change in overall suprathreshold spike response could be attributed to a change in the area and peak of the wPSP. This suggests that the LII/III to V pathway controls a steady excitation to LV following whisker stimulation over at least 50 ms (see Figures 5 and S1) and that depression of this pathway leads to a decrease and potentiation to an increase in spiking response. A lower level of principal whisker response occasionally occurred in the IB cells too, which was not seen in the LSPS studies and was manifest in the wPSP as a decrease in the slope. This is most likely explained by depression at an earlier synapse in the pathway. Consistent with this, after 10 days of deprivation, the LII/III responses are themselves depressed, most likely due to depression in the LIV to LII/III pathway (Allen et al., 2003; Glazewski and Fox, 1996).

Our LSPS studies revealed similar changes in other intracortical pathways in response to stimulation too, suggesting that the orthogonal response to deprivation characteristic of RS and IB cells is a general property of intracortical pathways. Recent studies have highlighted the importance of the LII/III to LV pathway in the barrel cortex for LV responses and plasticity. Blocking LII/III responses can prevent LV cells from spiking in response to principal whisker stimulation (Wright and Fox, 2010). During development, growth of the LII/III axons projecting to LV are sensitive to whisker trimming (Bruno et al., 2009) and could contribute to the plasticity described here if this form of anatomical plasticity is maintained into adulthood. Interestingly sensory deprivation also affected the ongoing activity in absence of stimulation, with an opposite effect in the two cell types. Such changes could emerge from a general increase of cortical inputs in IB cells and decrease in RS cells, as we observed ex vivo.

### Relative Contribution of Subcortical Pathways to Receptive Field Plasticity

Changes in cortical excitatory circuits are sufficient to account for the experience dependent changes in action potential rate across experimental methodologies. However, an increase in the short latency component of the spike response in RS cells corresponding to an increase in slope of the wPSP requires an additional factor to be introduced and one that could not be detected in the cortical photostimulation studies. The most likely candidates are therefore thalamic inputs and/or cortical inhibition. To consider thalamic inputs first, direct thalamic inputs to LV pyramidal neurons are known to exist (Bureau et al., 2006; Petreanu et al., 2009; Wimmer et al., 2010; Wright and Fox, 2010) and transmit fast enough to explain changes in the initial slope of the wPSP. The VPM nucleus provides the shortest latency synaptic drive to most S1 cortical neurons and therefore, an increase or decrease in the direct VPM input to a LV cell might be expected to change the initial component of the EPSP several milliseconds before polysynaptic intracortical inputs take effect. Many IB cells do not receive direct VPM inputs whereas most RS cells do (Agmon and Connors, 1992; Baranyi et al., 1993), which is consistent with the greatest changes in the initial component of the wPSP occurring in RS cells. VPM inputs (Wimmer et al., 2010) may also underlie the LVb depression observed in vivo 3 days after trimming, when intracortical circuits haven't yet depressed. Inhibition can also be activated early in the PSP by thalamic input (Gil and Amitai, 1996) and so could also conceivably be involved in modulating the amplitude of the direct thalamic drive to the cells. Inhibition could have a differential impact on RS and IB cells since RS cells are thought to receive more inhibitory control than IB cells (Schubert, Staiger et al., 2001). Testing which of these pathways are involved will require specific further studies.

### Classification of Pyramidal Cell Types

For practical reasons we were obliged to use different methods to classify RS and IB cells in the in vivo and ex vivo aspects of the study. Classically, injection of current at threshold elicits a complex of action potentials in IB cells and a single action potential in RS cells, which is sufficient to classify them (McCormick et al., 1985) and this method was used in the ex vivo studies. In the in vivo studies, we used this method in addition to identifying the action potential (AP) complexes that are characteristic of IB cells where the APs decrease in amplitude and ride upon a slow depolarization envelope (Baranyi et al., 1993; Connors et al., 1982; Dégenètais et al., 2002; McCormick et al., 1985; Nuñez et al., 1993) but see Nowak et al. (2003) for a quantitative analysis. Both methods are judged to be equivalent and indeed, in support of this view we described in the Experimental Procedures section different post hoc tests confirming that RS and IB cells have comparable properties in both our in vivo and ex vivo studies. However, the following qualifications need to be kept in mind: Schwindt and colleagues have reported high threshold bursting neurons that do not fire bursts at threshold current injections for producing APs (Schwindt et al., 1997; Steriade et al., 1993); such neurons would likely be included in the bursting neuron population in vivo but not ex vivo. Conversely, while slow depolarizations in vivo can be generated intrinsically they can also result from the activity of synchronized inputs, as occurs during the generation of up-states. Moreover, synaptic activity, release of neuromodulators in vivo or differences in physiological temperature can preclude or obscure the occurrence of bursts in IB cells (Wang and McCormick, 1993; Waters, 2011; Steriade, 2001; Steriade et al., 1993). However, the number of cells misclassified as a result of these confounds appears to be negligible based on our post hoc test of RS and IB cell properties (capacitance, morphology, bimodality of interspike intervals and laminar distribution—see Experimental Procedures). Therefore, the main observation that potentiation is restricted to IB cells and depression is restricted to RS cells holds for both in vivo and ex vivo data.

### Comparability of Rat and Mouse Studies

Our LSPS experiments were performed in mice while the in vivo intracellular recordings were performed in rats. Could the species difference alter the comparability of the results? It is possible that slight quantitative differences might be species-related, but the main qualitative result does not appear to be. When we repeated the extracellular receptive field study in mice we observed the same evolution of receptive fields across the different layers following deprivation. The main difference between plasticity in mice and rats was that potentiation occurred in LVa in rats but not in mice. One possible explanation for this would be the presence of fewer IB cells in LVa of mice. Some laboratories have reported a clear layer separation of thick tufted and thin slender cells in S1 (Groh et al., 2010; Meyer et al., 2010). In other studies, including the present one, thin slender regular spiking neurons were observed in LVb (Schubert et al., 2007). At the very least, all studies so far conclude that the distributions of pyramidal neuron types are not uniform throughout LV. Therefore, it is reasonable to hypothesize that the differences observed extracellularly between LVa and LVb result in part from differences in the percentage of RS and IB cells. If most cells in LVa of the mouse are of the RS type, we would not expect to see potentiation from the extracellular studies and indeed we do not. If LVa in the rat contains a mixture of RS and IB cells, as we found from our classification, then one would expect to see potentiation from the extracellular studies, which is the case.

### Cell-Type-Dependent Mechanisms for Orthogonal Plasticity

Synaptic plasticity varies with layer in sensory cortices, a factor that might be explained by the different connections within each layer (Wang and Daw, 2003). Synaptic plasticity affects receptive field organization both in supra- and infragranular barrel cortex neurons (Jacob et al., 2007). However IB cells and RS cells, which we show in this study to be differently potentiated during deprivation, share the same layer and largely the same connections including input from LII/III neurons. What then could be the mechanisms that drive their distinct forms of experience-dependent plasticity? The basal level of activity differs between RS and IB cells (de Kock et al., 2007), IB cells having larger spontaneous and evoked activity. Postsynaptic spike pattern and frequency influences the sign and amplitude of synaptic plasticity in vitro in cortical LII/III (Froemke et al., 2006; Zilberter et al., 2009) and LV pyramidal cells (Birtoli and Ulrich, 2004; Letzkus et al., 2006). This suggests that IB cells may be more plastic due to their propensity to fire more frequently and/or with bursts of action potentials. Back-propagation of action potentials into the dendritic tree associated with increased calcium influx has been hypothesized to play a major role in plasticity (Colbert, 2001; Sourdet and Debanne, 1999) and differs qualitatively between RS and IB cells (Grewe et al., 2010).

The parallels between structural spine plasticity and receptive field plasticity are remarkable. They have similar time course (Trachtenberg et al., 2002; Figures 6 and 7), express themselves predominantly in the same cell types (Holtmaat et al., 2006; Figure 3), and depend on the same signal transduction mechanisms (Wilbrecht et al., 2010). These similarities suggest strongly that the growth of new spines and associated synapse formation underlies receptive field plasticity (Knott et al., 2006). It remains to identify the presynaptic partners to these spine changes. Our studies strongly implicate LII/III to V projections and thalamic inputs as major candidates for future studies.

## Experimental Procedures

### Subjects and Whisker Deprivation

In vivo recordings were performed at Cardiff University and were approved under the UK Scientific Procedures Act 1986. C57Bl/6HsdOla mice and Long-Evans rats of both sexes were used for extracellular recordings (control: 7 rats and 9 mice; 3 day deprivation: 8 rats and 8 mice; 10 day deprivation: 10 rats and 8 mice). Intracellular recordings were performed in 23 control and 18 deprived Long-Evans male rats. In addition, 7 animals were required for histology only. The LSPS ex vivo study was performed on C57Bl/6J male mice at Cold Spring Harbor Laboratory, was approved by the Cold Spring Harbor Laboratory animal care and use committee and followed National Institutes of Health guidelines.

Subjects were lightly anesthetized with isofluorane and had either the left C or D row of whiskers trimmed to length <1 mm (same length as the fur hairs) every 24 or 48 hr. For LSPS ex vivo; control animals were anesthetized and handled in the same way as the deprived groups but their whiskers were left intact; whisker trimming started at postnatal day (P) 30 and was continued for 3 days or 10–14 days before the recordings. For in vivo recordings; whisker trimming started at postnatal day (P) 32–45 and was continued for 3 days or 10 days before recording; the trimmed whiskers were kept and glued to the whisker stump before stimulation. Control and deprived animals were recorded at the same age, i.e., P40–44 for ex vivo and P42–55 for in vivo. For mouse cortex, we found no difference in response levels for normal mice and those where we trimmed the whiskers and immediately reattached them in layers II/III, IV, Va, or Vb (ANOVA, effect of layer F(3,3) = 2.7, p = 0.045; gluing F(1,1) = 0.32, p = 0.56; interaction F(3,3) = 1.0, p = 0.37)) and similarly for rats where we only sampled in layers Va and Vb (ANOVA, effect of layer F(1,1) = 0.78, p = 0.38; gluing F(1,1) = 0.53, p = 0.47; interaction F(1,1) = 0.94, p = 0.34).

### In Vivo Recordings

#### In Vivo Surgery and Recording Procedures

Anaesthesia was induced with isoflurane and maintained with intraperitoneal injection of urethane (1.5 g/kg body weight). Anesthetic depth was monitored by observation of reflexes and breathing rate. As required, additional doses of urethane were injected to maintain anesthesia (0.15 g/kg body weight). Body temperature was maintained at 37°C. Specific procedures for extracellular recordings are described in Supplemental Experimental Procedures. For intracellular recordings, a 1 mm diameter craniotomy was performed over the D1–2 barrels. A separate craniotomy was made caudally away from the barrel field in order to insert a carbon fiber reference electrode at the cortical surface. Glass micropipettes filled with 1M potassium acetate and 2% byocytin (50–100 MΩ) were inserted in the brain through a small opening of the dura. Recordings were performed in current-clamp mode and the bridge was balanced manually (Axoclamp 2B). Electrode capacitance was compensated and no holding current was applied. Recordings with a membrane potential to action potential peak amplitude of less than 50 mV were excluded from the analysis. Between each stimulation sequence, a short hyperpolarizing current (10 pA, 100 ms) was injected in the cell and the series and membrane resistance were calculated through a double exponential fit. Four cells with an abnormal resistance were discarded (double exponential fit failed) and 7 cells (10% of total) with a low resistance for in vivo sharp recordings (<15 MΩ) were included.

#### In Vivo Whisker Stimulation

For extracellular recordings, whiskers were trimmed to similar lengths and stimulated with a 200 μm deflection from a piezoelectric stimulator positioned 10 mm from the follicle. The principle whisker and all of the immediate surrounding neighbor whiskers were consecutively stimulated with fifty ventrodorsal deflections at 1 Hz. For intracellular recordings, whiskers were deflected using nine independent computer-controlled piezoelectric actuators (Physik Instrument, UK) arranged in a bespoke frame (Manufacturing Engineering Centre, Cardiff University) designed to preserve the resting angle of each whisker, similar to a previous study (Jacob et al., 2010). Piezoelectric actuator movement was controlled by a 9 whisker stimulator (3901, CED UK). The deflection amplitude of each actuator was calibrated with a laser displacement-measuring system (Micro-Epsilon, Germany). Receptive fields were mapped with sparse noise stimulations composed of pseudorandom sequences of ventrodorsal deflections at 5 Hz (including a nonstimulation event). Five to one hundred twenty-five sequences (mode 50) were considered depending on the stability of the recording. The deflection lasted 30 ms (with 10 ms plateau) to avoid oscillations and were of 300 μm amplitude (see Figure 2 Jacob et al., 2010).

#### Analysis of In Vivo Data

All data were collected and analyzed using a CED1401 and Spike2 software (CED, UK). Action potentials (a.p.) were counted during 3 to 53 ms after stimulation unless specified. Spontaneous activity was subtracted (50 ms before stimulation for extracellular recordings, 3–53 ms after the nonstimulation event for intracellular recordings). Average latency and jitter (standard deviation) of the first action potential after stimulation in the 3–53 ms period after stimulation was calculated for the intracellular recordings. To measure the subthreshold activity, spikes were detected using the wavemark tool of the Spike 2 software and subtracted from the membrane potential trace (see Figure S5 for an evaluation of the effect). Whisker-evoked postsynaptic potentials (wPSP) were then averaged and latency, initial slope, amplitude of the first peak and area of the positive phase were calculated (see Supplemental Experimental Procedures for the details of the calculation).

#### Estimation of the Depth of In Vivo Recordings

Functional and histological methods were used to confirm that recordings were performed in a deprived whisker-related column (see Supplemental Experimental Procedures). Depths of layer borders were estimated independently for extracellular and intracellular recordings. Surface of liquid and subdural position, respectively, were chosen as references for sharp and carbon fiber electrodes. For extracellular recordings, we found LII/III between 0 and 270 μm, LIV to be 270–440 μm, LVa to be 440-550 μm and LVb to be 550-750 μm from the pia in mice. In rats we found LII/III between 0 and 470 μm, LIV to be 470–750 μm, LVa to be 750–1000 μm and LVb to be 1000–1250 μm from the pia. For intracellular recordings LV lays between 950 and 1400 μm from the surface of the saline solution above the pia.

### Ex Vivo LSPS Input Maps

Brain slices of the barrel cortex and whole cell recording were obtained as described (Shepherd and Svoboda, 2005), with minor modifications (see Supplemental Experimental Procedures). After whole-cell recording was established, the objective lens was switched to 4× (0.16 NA; UPlanApo, Olympus) and the stage was moved to align the barrel grid with respect to the LSPS stimulus pattern. LSPS was performed as described (Bureau et al., 2004; Shepherd et al., 2003; Shepherd and Svoboda, 2005). Briefly, stimulation with an ultraviolet laser (DPSS Lasers) was set on a 16 × 16 grid pattern spaced by 75 μm, covering 1.2 mm^2^ of cortex. This area included the entire thickness of the cortical gray matter and three barrel columns. NI-glutamate was uncaged for 1 ms with 30 mW of laser power at the specimen plane. We verified that under our experimental conditions these stimulation parameters elicited action potentials only when the laser beam was close to the soma of the neurons (Figure S3). Only excitatory inputs were mapped as cells were held at –65 mV, close to the reversal for fast inhibition. After the recordings the apical dendrites were imaged using fluorescence microscopy. Only the maps of cells where the apical dendrite ran parallel to the slice surface were included in the analysis.

For statistical analysis, we calculated the mean pixel values within a region of interest of the map for each individual cell, ignoring pixels that had direct responses. Values for different groups of cells were compared using the Wilcoxon rank-sum test. Significance is denoted as ^∗^p < 0.05, ^∗∗^p < 0.005, and ^∗∗∗^p < 0.0005. Data are presented as mean ± SD. Error bars in all plots denote standard errors of the mean.

### Cell Type Classification

Firing patterns in response to current injection were used to classify the recorded cells as RS or IB both for in vivo and ex vivo intracellular recordings (Connors and Gutnick, 1990; Schubert et al., 2001). For LSPS ex vivo (Figures 6B, 6C, and 6D), cells firing high frequency bursts of action potentials at threshold (including doublets; first interspike interval (ISI) <25 ms, mean frequency 99 ± 28 Hz) were classified as IB (Schubert et al., 2001; Schwindt et al., 1997). Cells firing a train of action potentials with spike frequency adaptation (first ISI > 25 ms, mean frequency 16 ± 9 Hz) were classified as RS (Figure 6D). This criterion could not be used for in vivo recordings since chronic firing rate at rest precluded stimulating at threshold. Indeed RS cells exhibited irregular activity reflecting spontaneous inputs and IB cells bursts occurred stochastically position after current injection. Firing patterns were classified as IB when a characteristic burst shape occurred at least once in response to current injection (Connors et al., 1982). The burst shape was defined as high frequency action potential decreasing in size at the top of a slow depolarization (“calcium”) event. Note LV bursts have a characteristic shape compared to other layers. To double-check our classification we compared it with the criterion for in vivo classification introduced by Nowak et al. (2003), i.e., bimodality of the distribution of log interspike interval (Figure S4). We calculated this during spontaneous activity and found a match between both methods for 88% of the cells. The few mismatches were often due to up & down state activity; occurrence of bursts with little sodium channel adaptation and no slow depolarization; or a sparse occurrence of bursts.

The different classification methods necessitated by the practicalities of in vivo and ex vivo experiments nevertheless segregated comparable populations of neurons as judged by several post hoc comparisons. First, IB cells had larger capacitance than RS cells both ex vivo (sum of rank test p < 0.0005) and in vivo (sum of rank test p < 10^−7^). Apparent membrane capacitance is known to be correlated with total membrane area and differs between thick tufted and thin slender pyramidal neurons (Larkman et al., 1992). Second, the distribution of the log inter spike interval was bimodal for IB cells and monomodal for RS cells both in vivo and ex vivo (Figure S4). Third, a subset of recorded cells was filled with biocytin. Ex vivo the dendrites of 7 RS cells and 7 IB cells were reconstructed for morphological analysis with Neurolucida software (Microbrightfield), and in vivo the dendrites of 25 IB cells and 8 RS cells were analyzed in 2-D with a Neurolucida camera. Among them 7 IB cells and 5 RS cells were used only for morphological analysis since no receptive field was recorded. In both experiments IB cells had thick apical dendrites with a dominant bifurcation in LII/III or LIV and an elaborate apical tuft (Chagnac-Amitai et al., 1990; Le Bé et al., 2007; Schubert et al., 2001) (Figures 2C and 6D). RS cells had a relatively thin apical dendrite and a small apical tuft branching close to the pia. Finally, ex vivo recordings were performed in LVb and most in vivo recorded IB cells were located in LVb as expected from the literature (Figure 2; Nowak et al., 2003). For further treatment of the validity and limits of the classification methods see the Discussion.

## Supplemental Information

Document S1. Figures S1–S6, Tables S1 and S2, Supplemental Discussion, and Supplemental Experimental Procedures
