# Supplementary material for: Regular Spiking and Intrinsic Bursting Pyramidal Cells Show Orthogonal Forms of Experience-Dependent Plasticity in Layer V of Barrel Cortex
Source: Neuron. 2012 Jan 26;73(2):391–404. doi: 10.1016/j.neuron.2011.11.034 (PMC3524456; doi:10.1016/j.neuron.2011.11.034)
Supplement: Document S1. Figures S1–S6, Tables S1 and S2, Supplemental Discussion, and Supplemental Experimental Procedures [file mmc1.pdf]

Neuron, Volume *73*

Supplemental Information

Regular Spiking and Intrinsic Bursting Pyramidal Cells Show Orthogonal Forms of Experience-Dependent Plasticity in Layer V of Barrel Cortex

Vincent Jacob, Leopoldo Petreanu, Nick Wright, Karel Svoboda, and Kevin Fox

Index of Supplemental Information

1. **Supplemental Figures**
   S1: Detailed expansion of the data presented in main Figure 4
   S2: Integrate and fire model to test whether the PSTH data and hence the plasticity observed can be predicted from the subthreshold measures of Vm. Relates to main Figure 5.
   S3: Methodological information relating to LSPS and related to main Figures 6,7,8.
   S4: Interval histogram data for RS and IB cells providing extra information relative to classification of the two cell types and therefore related to main Figures 2 and 6.
   S5: Validation of the spike removal procedure used for the in vivo intracellular analysis of wPSPs and therefore related to main Figures 3 and 4.
   S6: Time course of intercolumnar RS and intracolumnar IB inputs from layer 2/3, compliments the significant changes show in Figure 8.
2. **Supplemental Tables**
   Documents in detail the post-hoc statistical analysis presented for the extracellular data in main Figure 1.
3. **Supplemental Experimental Procedures**
   Relates to standard procedures used for the 3 groups of experiments.
   Relates to the modelling methods in supplemental Figure 2.
4. **Supplemental Discussion (methodological issues)**

Relates to Figures 2 and 6 and the methods used by us and others in the literature for classifying cells as RS or IB.

5. Supplemental References

References for the supplemental text.

1. Supplementary Figures

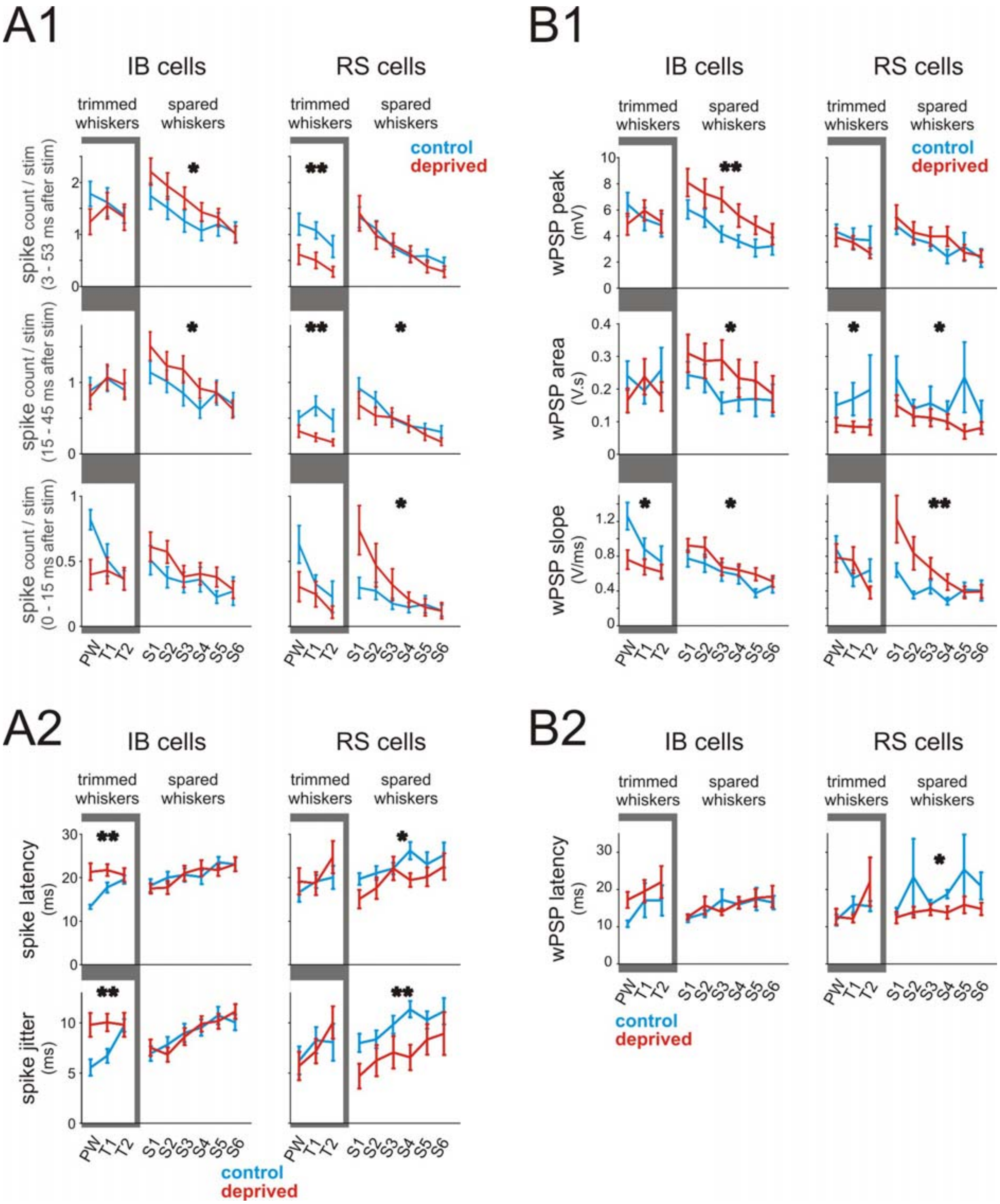

Supplementary Figure 1: Effect of deprivation detailed for all the whiskers measured in the receptive fields. A. Suprathreshold spatial (A1) and temporal (A2) receptive fields in control (blue) and 10 day deprived (red) animals. Average spike rate, spike latency and spike jitter are shown for each individual whisker measured in the receptive fields. For each cell, PW is the principal whisker (trimmed), T1 and T2 are the surround whiskers in the row that were also trimmed and S1 to S6 are the spared whiskers in the adjacent rows (C and E). Responses are sorted by decreasing suprathreshold spike rate. The unit for the spike rate (spk/50 stim) is equivalent to the unit for suprathreshold response (spk/stim), since the window of integration is 50 ms. Statistical significance is calculated using an ANOVA, looking at the effect of deprivation on all spared or all trimmed whiskers (* $p<0.05$, ** $p<0.001$). **B.** Subthreshold spatial (B1) and temporal (B2) receptive fields in control and deprived animals. Average whisker evoked PSP amplitude, area, initial slope and latency are shown for each whisker. Trimmed and spared whiskers are sorted by decreasing suprathreshold response rate. Other conventions are the same as in **A**.

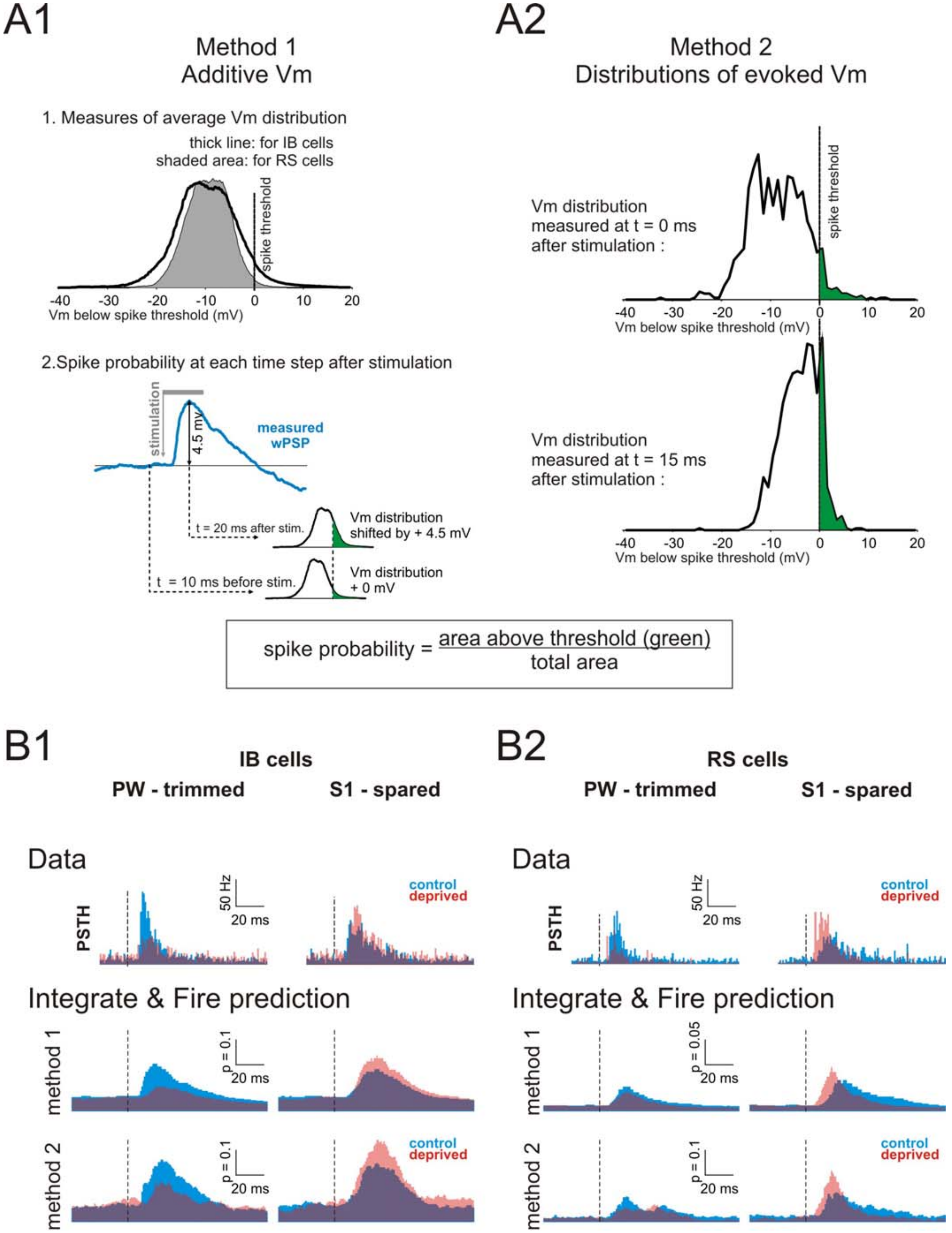
A1
Method 1
Additive Vm
1. Measures of average Vm distribution
thick line: for IB cells
shaded area: for RS cells
spike threshold
Vm below spike threshold (mV)
2.Spike probability at each time step after stimulation
stimulation
4.5 mV
measured wPSP
t = 20 ms after stim.
Vm distribution shifted by + 4.5 mV
t = 10 ms before stim.
Vm distribution + 0 mV
A2
Method 2
Distributions of evoked Vm
Vm distribution measured at t = 0 ms after stimulation :
Vm distribution measured at t = 15 ms after stimulation :
spike threshold
Vm below spike threshold (mV)
spike probability = area above threshold (green) / total area
B1
IB cells
PW - trimmed
S1 - spared
B2
RS cells
PW - trimmed
S1 - spared
Data
PSTH
50 Hz
20 ms
control
deprived
Integrate & Fire prediction
method 1
method 2
p = 0.1
p = 0.05
20 ms

Supplementary Figure 2: Plasticity of suprathreshold activity is predicted by a simple Integrate & Fire model applied to subthreshold activity. A. For a simple Integrate & Fire (I&F) model, the probability of evoking a spike is equal to the probability that the membrane voltage (Vm) is above spike threshold. Two methods based on this principle were calculated to fit a simple I&F model to subthreshold Vm fluctuations. **A1.** First method: hypothesising that stimulation induces an additive depolarisation to the distribution. Upper panel; for each cell Vm was aligned to the measured spike threshold and the Vm distribution calculated across the cell population. Since the variance of the (Vm) amplitude distribution was larger for IB cells than for RS cells, we use separate distributions for each. Lower panel; the cell averaged wPSP was calculated for each cell type and condition (see Figure 5). For each time step around stimulation, the average Vm distribution is shifted by the amount of depolarisation measured from the wPSP. Since the spike threshold is fixed the probability of spiking varies. Since wPSP are averaged per cells, method 1 can be compared directly with the raw data in main Figure 5. **A2.** Second method: the Vm amplitude distribution is directly calculated at each time step around stimulation from the evoked responses of all the cells. This method models the data more closely and takes into account individual cell's Vm distribution: note that in the example, the variance is lower during evoked activity (lower panel) compared to spontaneous activity (upper panel). **B.** Measured (upper panel) and predicted (lower panels) PSTHs in response to stimulation of principal (PW) and best spared (S1) whiskers. IB cells are known to have complex intrinsic properties, accordingly the shape of PSTHs are not properly predicted by a simple I&F model. Nevertheless, direction and time-courses of the changes induced by deprivation are similar to measured PSTHs, including the combined early potentiation and late depression in response to RS cells – spared whiskers stimulations. In conclusion, Vm fluctuations reflecting synaptic activity are sufficient to explain the observed plasticity.

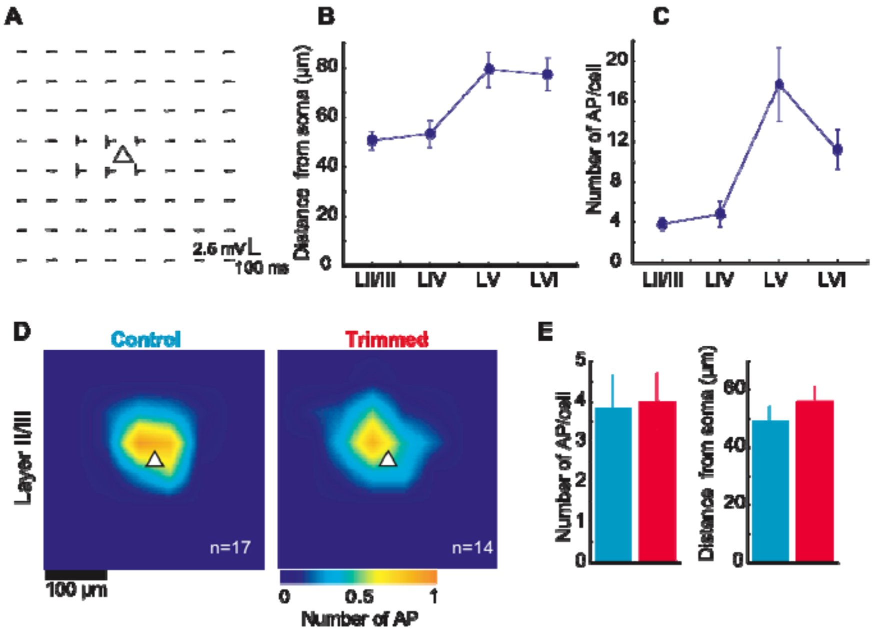

Supplementary Figure 3: Excitation profiles and resolution of photoactivation by glutamate uncaging.

A. Example of the excitation profile for a pyramidal cell in LII/III. Traces (50 µm spacing) were acquired in loose patch mode. **B.** Mean distance from the soma at which action potentials (APs) were elicited in each cortical layer (calculated as Σ(AP x distance from soma)/ Σ(AP)). (n=9-15 per layer) **C**. Total number of APs per cell per map for each cortical layer. (n=9-15 per layer) **D**. Average excitation profiles of LII/III pyramidal cells in control animals or in deprived barrel columns. Colors represent the mean number of action potentials (APs) recorded in loose-patch mode within 100 ms after photostimulation. The positions of the somata are indicated by white triangles. **E,** Total number of APs per cell per map and mean distance from the soma at which APs were elicited in control (blue) and deprived barrel columns (red).

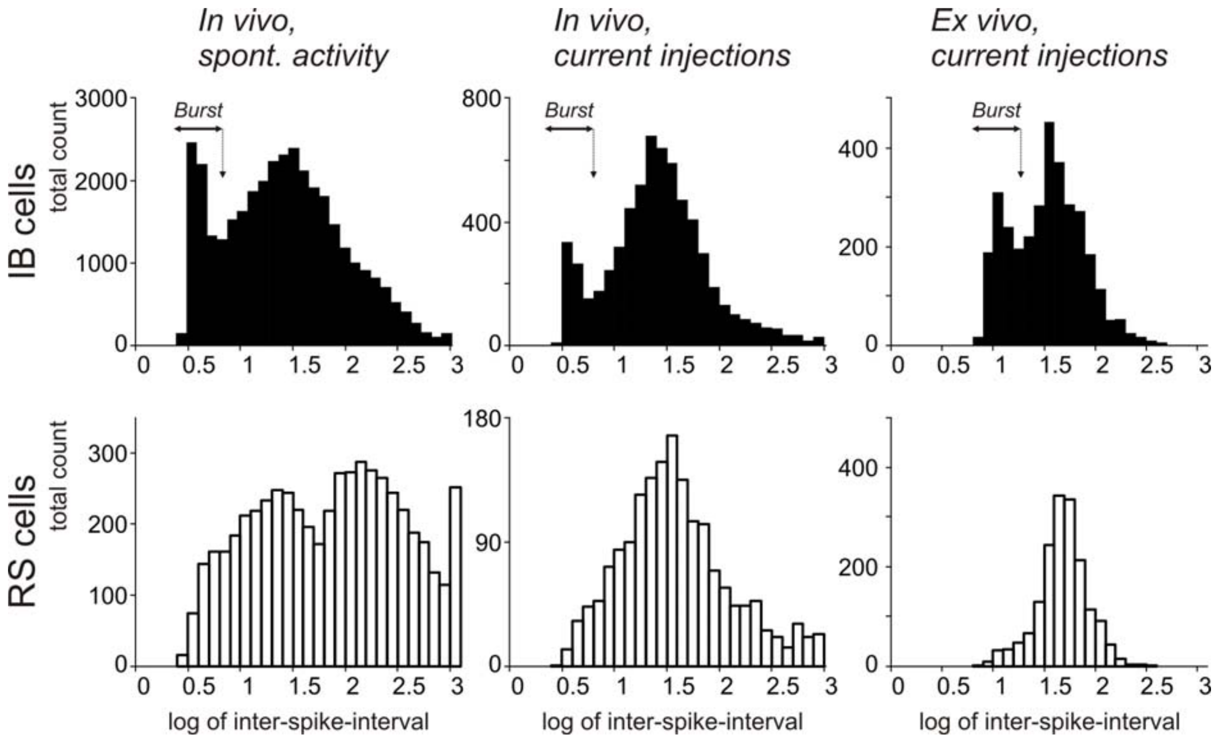

Supplementary Figure 4: Distribution of log inter-spike-interval for RS and IB cell populations.

The distribution of log inter-spike-interval for IB (black) and RS (white) populations are shown by cell type and recording condition for intracellular (*in vivo*) spontaneous activity and during current injection and for whole cell patch clamp (*ex vivo*) recordings. The spontaneous activity recorded *in vivo* corresponds to period without whisker stimulations or current injection. Current injections consists of 500 ms long steps of varying amplitude. *Ex vivo*, spikes occurred only during current injection periods. Note that the frequency of bursts is higher *ex vivo* compared to *in vivo*, probably due to different recording temperatures.

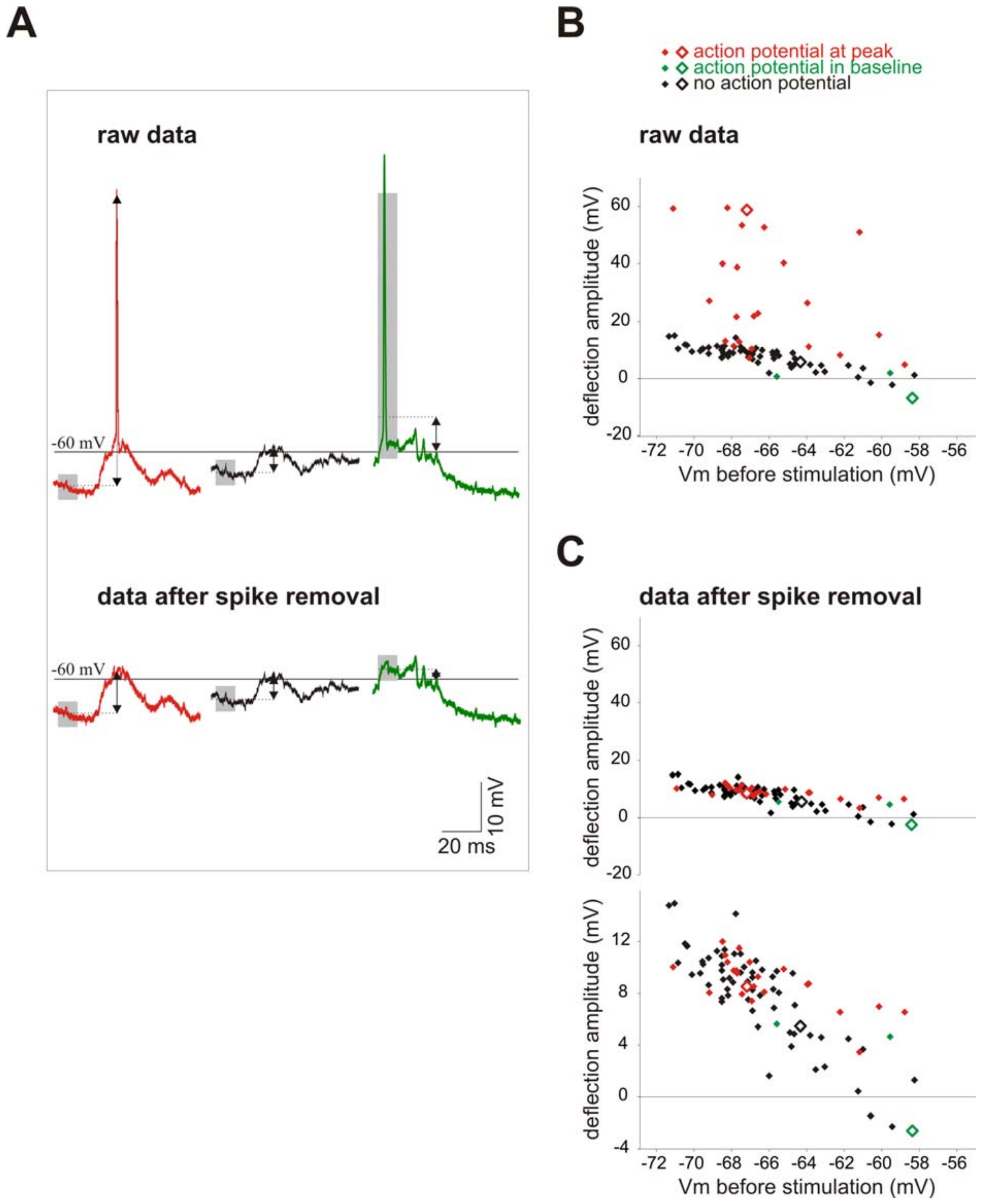

Supplementary Figure 5: Effect of the spike removal procedure on the wPSPs

This figure presents responses to individual stimulations of the principal whisker for a single cell recorded intracellularly *in vivo*. **A**. Three examples of voltage membrane

fluctuation in response to single deflections. Top row, original data; bottom row, data after spike removal. The baseline periods are indicated by the grey shaded area and are measured as an average over the 10 ms period before whisker stimulation (dashed grey line). Note that a spike can occasionally occur spontaneously in the baseline period (green). The deflection amplitude (double headed arrow) plotted in panels **B** and **C** is calculated at the time of the peak averaged wPSP. **B.** Scatterplot showing the deflection amplitude with spikes present as a function of baseline Vm. Open diamonds shows the three example traces from panel **A**. Trials including a spike are shown in red and those without are shown in black. **C**. Scatterplots for deflection amplitude versus baseline Vm after spikes have been removed from the data. Conventions the same as panel **B**. The top and bottom scatterplots show the same data, except the vertical scale is enlarged in the bottom scatterplot. Note that the wPSP amplitudes with spikes removed (red) lie in the same range as the wPSP amplitudes that naturally occurred without a spike (black) and that this is the case across the whole range of baseline Vm.

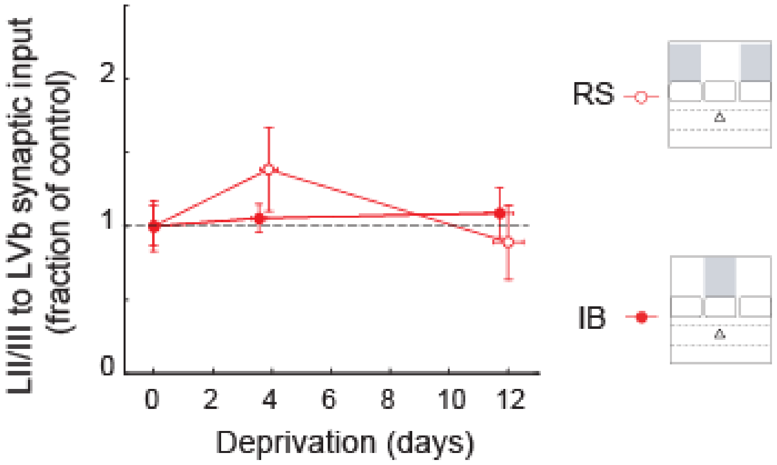

Supplementary Figure 6: Time course of the LII/II to LVb RS spared barrel input and the LII/III to L5b IB home barrel input .

Time course of LII/III to LVb RS input from the surround columns (open circles), and LII/III to LVb IB input from the home barrel (filled circles), normalized to controls.

2. Supplementary Tables

RAT

Deprivation	Layer	Animals	Cells	PW	S1	S2
0	II/III	4	33			
3	II/III	7	37	No change<br>t(68)=0.54 p=0.59	No change<br>t(68)=1.75, p=0.08	No change<br>t(68)=1.45, p=0.15
10	II/III	9	23	Decreased<br>t(54)= 3.1,p<0.005	Decreased<br>t(54)=2.61, p<0.05	Decreased<br>t(54)=2.7, p<0.01
0	IV	4	29			
3	IV	8	51	No change<br>t(78)=0.49, p=0.62	No change<br>t(78)=1.6, p=0.10	No change<br>t(78)=0.98, p=0.33
10	IV	9	40	No change<br>t(67)=0.3, p=0.76	No change<br>t(67)=1.0, p=0.3	No Change<br>t(67)=0.88, p=0.38
0	Va	7	22			
3	Va	8	48	No change<br>t(68)=0.89, p=0.38	Increased<br>t(68)=3.17, p<0.005	Increased<br>t(68)=3.01, p<0.005
10	Va	10	40	No change<br>t(60)=0.98, p=0.33	Increased<br>t(60)=2.95, p<0.005	Increased<br>t(60)=3.0, p<0.004
0	Vb	3	37			
3	Vb	8	36	Decreased<br>t(71)=5.3, $p<10^{-3}$	Increased<br>t(71)=5.1, $p<10^{-3}$	Increased<br>t(71)=4.9, $p<10^{-3}$
10	Vb	9	56	Decreased<br>t(91)=8.3 $p<10^{-3}$	Increased<br>t(91)=4.0, $p<10^{-3}$	Increased<br>t(91)=4.2, $p<10^{-3}$
		TOTAL	452			

Supplementary Table 1. Rat data: Statistical analysis of extracellular recordings from the rat subdivided by layer and deprivation period. The number of animals contributing to each sample of cells is shown for each layer and deprivation period. Zero indicates control animals without any deprivation. The result of statistical comparison between control and 3 or 10 days deprivation is shown for the principal whisker (PW), the strongest surround whisker (S1) and the second strongest surround whisker (S2). Statistically significant decreases in the mean response are highlighted in red and increases in green.

MOUSE

Deprivation	Layer	Animals	Cells	PW	S1	S2
0	II/III	6	59			
3	II/III	4	70	No change t(127)=0.35 p=0.73	Decreased t(124)=5.6 p<10-3	Decreased t(122)=5.1 0<10-3
10	II/III	7	37	Decreased t(94)=4.2 $p<10^{-3}$	No change t(91)=0.12 p=0.9	No change t(89)=0.02 p=0.98
0	IV	9	45			
3	IV	8	35	No change t(78)=1.7 p=0.09	No change t(78)=1.6 p=0.11	No change t(78)=1.12 p=0.26
10	IV	8	44	No change t(87)=1.3 p=0.18	No change t(87)=0.08 p=0.93	No change t(87)=0.57 p=0.57
0	Va	6	22			
3	Va	6	20	Decrease t(40)=2.3 p<0.03	No change t(40)=-.14 p=0.89	No change t(40)=0.31 p=0.76
10	Va	5	11	Decrease t(31)=3.7 $p<10^{-3}$	No change t(31)=0.38 p=0.70	No change t(31)=0.42 p=0.68
0	Vb	6	53			
3	Vb	8	47	Decreased t(98)=2.5 p<0.02	Increased t(98)=4.1 p<10-4	Increased t(98)=3.9 $p<2x10^{-4}$
10	Vb	6	31	Decreased t(82)=3.4 p<0.002	Increased t(82)=2.3 p<0.03	Increased t(82) =2.2 p<0.03
		TOTAL	474			

Supplementary Table 2. Mouse data: Statistical analysis of extracellular recordings from the mouse subdivided by layer and deprivation period. The number of animals contributing to each sample of cells is shown for each layer and deprivation period. Zero indicates control animals without any deprivation. The result of statistical comparison between control and 3 or 10 days deprivation is shown for the principal whisker (PW), the strongest surround whisker (S1) and the second strongest surround whisker (S2). Brown indicates cases where the rat and mouse differ. Blue indicates that the effect of deprivation is the same between mouse and rat.

3. Supplementary experimental procedures

Extracellular In vivo surgery and recording procedure

The animal was placed in a stereotaxic frame. For extracellular recordings, the skull was thinned over the barrel field until transparent and flexible. A small hole was made with a hypodermic needle for each penetration to allow a glass-insulated carbon fiber microelectrode into the cortex. Extracellular spikes were recorded at a bandwidth of 600 Hz to 6 kHz and sorted online using a dual threshold spike discriminator (Neurolog). The principal whisker and the 8 immediate neighbour whiskers were trimmed to 12 mm length and inserted 3 mm into short tubes glued on the actuator.

Analysis of in vivo data. We calculated suprathreshold peri-stimulus timing histograms (PSTH) and subthreshold whisker-evoked post-synaptic potentials (wPSP). The derivative of the average wPSP (with a 3 ms long time constant) was calculated. Latency was considered as the first time point when the derivative crossed a threshold of mean $\pm$ 3 standard deviation of the spontaneous activity. When the threshold was not crossed but a wPSP was apparent, the latency was defined as the time at which the maximum of the first order time derivative occurred (1.4 % of the cases). Occasionally a false positive was obvious and in these cases we used time at which the derivative crossed the threshold for the second time (3.8 % of the cases). The latency was not calculated in the absence of an obvious wPSP. Initial slope was defined as the maximum of the first order time derivative in the 4 ms time window after the start of the response. The amplitude of the first peak was calculated by subtracting the average level of the baseline (measured over a period of 10 ms preceding stimulation). If not specified otherwise in the text, the area of the positive phase of the wPSP was defined to occur between the stimulus onset and the time when the peak decreased back to 10% of its maximum amplitude.

Estimation of the recording position for in vivo recordings. We checked that each recording was performed in a deprived whisker-related column. For extracellular recordings, the microelectrode penetration was marked by a lesion in LIV (1 μA, 10 s tip negative) at the end of recording in each penetration. Subjects were perfused and horizontal sections of barrel field were reacted for cytochrome oxidase activity to

visualize the barrel field pattern of LIV. This permitted accurate confirmation of horizontal location and depth of individual recordings. Additional coronal sections confirmed the depths of the different layers. For intracellular recordings, the horizontal location was obtained from the receptive fields of local field potentials recorded with the micropipettes electrodes in LIV before impaling the cell (Figure 2). In a subset of experiments the electrode was not perpendicular to the brain surface and the angle was corrected for calculating depth. After recordings animals were perfused and byocytin staining was vizualised in coronal sections (300 μm thick). Laminar position was confirmed whenever possible and LV found to lie between 950 to 1400 μm from the surface of the saline solution above the pia.

Ex vivo laser scanning photo-stimulation

Slice preparation.

Slices were prepared as described (Shepherd and Svoboda, 2005), with minor modifications. Animals were anesthetized with a an intraperitoneal injection of a ketamine/xylazine mixture (0.13 mg ketamine/0.01 mg xylazine/g body weight) and perfused through the heart with a small volume of ice cold ACSF containing in mM (in mM): 127 NaCl, 25 NaHCO3, 25 D-glucose, 2.5 KCl, 1 MgCl2, 2 CaCl2, and 1.25 NaH2PO4, aerated with 95% O2/5% CO2. The brain was removed and placed in an icecold cutting solution containing (in mM): 110 choline chloride, 25 NaHCO3, 25 Dglucose, 11.6 sodium ascorbate, 7 MgCl2, 3.1 sodium pyruvate, 2.5 KCl, 1.25 NaH2PO4, and 0.5 CaCl2. 300 μm thick slices of the right barrel cortex, orthogonal to the barrel rows, were cut with a Microm (Walldorf, Germany) vibrating slicer and incubated in oxygenated ACSF for 45 min before the recordings. To correctly identify individual barrel rows we only used slices in which 5 large barrels, corresponding to each of the 5 whisker rows, were clearly distinguishable (Allen et al., 2003; Finnerty et al., 1999) (Fig.6A). Pyramidal cells in LVb were recorded up to 140 μm below the Va/Vb border.

Ex vivo electrophysiology

Slices were continuously perfused at room temperature with ACSF containing 5μM CPP (Tocris,Ellisville, MO) and 0.37 mM NI-glutamate (Sigma-RBI, St. Louis, MO). Neurons were visualized under infrared differential interference optics and patched with borosilicate pipettes (resistance 4-6 MΩ). The intracellular solution contained in mM (in mM) 120 Kgluconate, 5 NaCl, 2 MgCl2, 0.1 CaCl2, 10 HEPES, 1.1 EGTA, 4 Mg2ATP, 0.4 Na2GTP, 15 sodium phosphocreatine, and 0.015 Alexa-594 (Molecular Probes) (pH 7.25; 290 mOsm). Cells were recorded at a depth of 50 to 95 μm. Recordings were obtained only from barrel columns in rows C and D. Immediately after breaking in cells were depolarized by injection of graded current pulses.

LSPS Analysis

Responses were analyzed within 100 ms after the UV stimulus. Direct and synaptic responses were separated according to their different onset time (Schubert et al., 2001; Shepherd et al., 2003). Responses with an onset time below 6 ms were categorized as direct (i.e. purely postsynaptic) and later responses as synaptic. In control experiments (data not shown) we confirmed that this time window allowed us to correctly identify more than 90% of the direct responses. Direct responses were detected in the perisomatic region and the apical dendrite, in many cases up to L1 (Fig. 6 E,F). Sites with direct responses were excluded from the calculations of averages and represented as black pixels (Fig. 6E). As most of the pixels over L5 and L4 in the home barrel were contaminated by direct responses due to the abundance of basal and apical oblique dendrites, we excluded those regions from analysis. Synaptic input maps were calculated as the mean current in a response window from 6 to 100 ms. Thus, pixel values represent synaptic charge. However, for consistency with previous studies and because synaptic current is a more familiar unit, data are shown in units of picoamperes. Background levels of synaptic input (dashed lines in Fig. 7 J,K), due to spontaneous synaptic activity, were calculated for each cell by analyzing the mean current during 100 ms before the UV stimulus. Typically 2 to 3 maps were obtained for each cell and averaged. Average maps for single cells were used to obtain group averages. For display purposes averaged maps were linearly interpolated. Layers 4 and 5 in the home barrel were excluded from the

analysis and blacked out from the averaged maps as most events were polluted by direct responses.

Excitation Profiles

To calibrate the resolution and intensity of LSPS as well as to control whether photoexcitability was altered with sensory deprivation we performed single-cell maps of action potentials (AP) elicited by LSPS (Shepherd et al., 2003; Shepherd and Svoboda, 2005) (Supplementary Figure 3) . Excitation profiles were measured under the same experimental condition than synaptic inputs maps except that neurons were recorded in loose-seal configuration in current clamp mode. Cells were stimulated with an 8 x 8 pattern centered on the soma, spaced by 50 μm. The number of APs elicited at each site up to 100 ms after the stimulus were counted. Most APs occurred with latencies <20 ms.

Integrate and Fire model.

We constructed two Integrate & Fire models as detailed below, both of which were fed by recorded subthreshold activity in order to predict a suprathreshold output and hence compose post-stimulus time histograms (PSTH). The predicted and measured PSTHs were then compared to check correspondence between the two levels of analysis.

For each cell we measured the membrane potential (Vm) at the spike threshold using spike-triggered-averaging. The spike threshold was taken as Vm just before the spike at the time of the peak of the second derivative of Vm (i.e. at the point of maximum rate of change of Vm during spike initiation). For both methods described below, the spike threshold voltage was subtracted from the recordings and consequently Vm was expressed relative to spike threshold.

Method 1: The amplitude distribution of Vm was measured separately for IB and RS cell populations (Supplementary Figure 2A1). The mean and SDs of the Vm amplitude distributions were respectively -9.7±6.4 mV and -9.5±4.3 mV (relative to spike threshold of zero). Spike probability was defined as the probability that Vm exceeded spike threshold, calculated from the area of the Vm amplitude distribution (area above 0 divided by total area). Any given sensory evoked depolarisation was added to the entire

Vm distribution, affecting its mean but not its SD. The area above threshold increased or decreased accordingly and spike probability was recalculated (Supplementary Figure 2A1). The average depolarisation amplitudes were measured for each time bin around the period of sensory stimulation, and corresponding spike probabilities calculated. The resulting values were depicted in histograms that could be directly compared to the real PSTHs (Supplementary Figure 2B).

Method 2:
For each time bin of the PSTH, the amplitude distribution of Vm was measured across the whole cell population (Supplementary Figure 2A2). As a consequence, the mean and SDs of the Vm amplitude distribution differed for each time bin. Spike probabilities were calculated as the probability that Vm was above spike threshold in the corresponding distribution (area above 0 divided by total area) and used to build the predicted PSTH (Supplementary Figure 2B).

Note that for each method, the spike threshold was measured rather than calculated and therefore could not be modified to fit the model to the data. Note also that we did not impose an arbitrary refractory period following generation of each spike and instead relied on the measured trajectory of Vm following spike production and hence its relationship to spike probability to take this into account automatically (in method 2). As can be seen from Supplementary Figure 2, the measured and predicted PSTHs have several features in common but are not identical. To solve the discrepancy between the predicted and real data, a more complex model would be required that includes conductances and a moving spike threshold.

4. Supplementary references

Allen, C. B., Celikel, T., and Feldman, D. E. (2003). Long-term depression induced by sensory deprivation during cortical map plasticity in vivo. Nat Neurosci *6*, 291-299.

Connors, B. W., Gutnick, M. J., and Prince, D. A. (1982). Electrophysiological properties of neocortical neurons in vitro. J Neurophysiol *48*, 1302-1320.

Finnerty, G. T., Roberts, L. S., and Connors, B. W. (1999). Sensory experience modifies the short-term dynamics of neocortical synapses. Nature *400*, 367-371.

Schubert, D., Staiger, J. F., Cho, N., Kotter, R., Zilles, K., and Luhmann, H. J. (2001). Layer-specific intracolumnar and transcolumnar functional connectivity of layer V pyramidal cells in rat barrel cortex. J Neurosci *21*, 3580-3592.
Shepherd, G. M., Pologruto, T. A., and Svoboda, K. (2003). Circuit analysis of experience-dependent plasticity in the developing rat barrel cortex. Neuron *38*, 277-289.
Shepherd, G. M., and Svoboda, K. (2005). Laminar and columnar organization of ascending excitatory projections to layer 2/3 pyramidal neurons in rat barrel cortex. J Neurosci *25*, 5670-5679.
